# Comparing Anxiety and Depression in Information Technology Workers with Others in Employment: A UK Biobank Cohort Study

**DOI:** 10.1093/annweh/wxac061

**Published:** 2022-08-27

**Authors:** Drushca Lalloo, Jim Lewsey, Srinivasa Vittal Katikireddi, Ewan B Macdonald, Desmond Campbell, Evangelia Demou

**Affiliations:** 1 Healthy Working Lives Group, Institute of Health and Wellbeing, University of Glasgow, Glasgow, UK; 2 Health Economics and Health Technology Assessment, Institute of Health and Wellbeing, University of Glasgow, Glasgow, UK; 3 MRC/CSO Social and Public Health Sciences Unit, Institute of Health and Wellbeing, University of Glasgow, Glasgow, UK

**Keywords:** information technology workers, anxiety/depression, mental health, UK Biobank, computer professionals

## Abstract

**Objectives:**

Despite reported psychological hazards of information technology (IT) work, studies of diagnosed mental health conditions in IT workers are lacking. We investigated self-reported mental health outcomes and incident anxiety/depression in IT workers compared to others in employment in a large population-based cohort.

**Methods:**

We evaluated self-reported mental health outcomes in the UK Biobank cohort and incident diagnosed anxiety/depression through health record linkage. We used logistic regression and Cox models to compare the risks of prevalent and incident anxiety/depression among IT workers with all other employed participants. Furthermore, we compared outcomes within IT worker subgroups, and between these subgroups and other similar occupations within their major Standard Occupational Classification (SOC) group.

**Results:**

Of 112 399 participants analyzed, 4093 (3.6%) were IT workers. At baseline, IT workers had a reduced odds (OR = 0.66, 95%CI: 0.52–0.85) of anxiety/depression symptoms and were less likely (OR = 0.87, 95%CI: 0.83–0.91) to have ever attended their GP for anxiety/depression, compared to all other employed participants, after adjustment for confounders. The IT technician subgroup were more likely (OR = 1.22, 95%CI: 1.07–1.40) to have previously seen their GP or a psychiatrist (OR = 1.31, 95%CI: 1.06–1.62) for anxiety/depression than their SOC counterparts. IT workers had lower incident anxiety/depression (HR = 0.84, 95%CI 0.77–0.93) compared to all other employed participants, after adjustment for confounders.

**Conclusions:**

Our findings from this, the first longitudinal study of IT worker mental health, set the benchmark in our understanding of the mental health of this growing workforce and identification of high-risk groups. This will have important implications for targeting mental health workplace interventions.

## Introduction

Information technology (IT) has advanced at an extraordinary pace. Compared to the rest of the UK economy, the IT industry grew six times faster than any other industry in 2019 contributing £149bn to the economy in 2018 (Tech Nation, 2020). Accordingly, the IT workforce has been growing more rapidly than others, now accounting for almost 10% of the UK workforce; a 40% increase from 2017 (Tech Nation, 2020; US Bureau, 2020).

Defining an IT worker is complex. Broadly, IT workers are a skilled occupational group who develop and maintain computer systems. They should be distinguished from other workers who use computers day-to-day as part of their jobs. IT roles are diverse and include data management, software, hardware and network design/development/management and helpdesk assistance (Freeman and Aspray,1999). Information/‘cyber’ security has become an important job function, as have artificial intelligence, robotics, virtual reality, and ‘big data’ collection/analyses for consumer profiling.

With integration of IT into our daily and working lives, IT jobs are now dispersed across multiple sectors (e.g. businesses, government, education, and healthcare) and are not exclusively located within the IT industry. This wide distribution makes identifying and studying IT workers challenging (Freeman and Aspray,1999).

Psychological hazards of IT work include: adverse working patterns and hours (particularly with globalization), increased workload, work demands and pace of work and interference with personal/family life (Rocha and Debert-Ribeiro, 2004; Hoonakker *et al.*, 2005; Tominaga *et al.*, 2007; Kivisto *et al.*, 2008; Sharan *et al.*, 2011; Das, 2012; Nayak, 2014). These in turn have been associated with work-related stress, burnout, mental ill-health, insomnia and high workforce turnover (Rocha and Debert-Ribeiro, 2004; Kouvonen *et al.*, 2005; Sharma *et al.*, 2006; Joseph *et al.*, 2007; Pawlowski *et al.*, 2007; Kivisto *et al.*, 2008; Rashidi and Jalbani, 2009; Zadeh and Begum, 2011; Darshan *et al.*, 2013; Shih *et al.*, 2013; Nayak, 2014; Padma *et al.*, 2015). The rapid advancement and increasing scope of IT, with a constant need to keep abreast of changes/developments and continuously upskill, creates added pressure (Nayak, 2014).

Equally however, IT workers are generally well-paid, least socio-economically deprived (Lalloo *et al.*, 2021) and IT work provides substantial worker autonomy and prospects of significant employment security (Freeman and Aspray,1999).

Stress/psychological issues are within the top three work-related conditions reported among IT workers (Pinto *et al.*, 2004; Rocha and Debert-Ribeiro, 2004; Sharma *et al.*, 2006). The extent and type of problems varies with job profile, with increased reports in software developers (Rashidi and Jalbani, 2009; Das, 2012; Nayak, 2014). A 2019 UK survey suggested that technology professionals experienced stress levels similar to healthcare workers, with 66% reporting work-related stress (BIMA, 2019). While stress in itself is not usually considered a medical condition, if prolonged or excessive it can result in mental ill-health, including anxiety and depression (HSE, 2020).

The adverse impact of common mental health conditions (i.e. anxiety/depression) are significant and far-reaching, not just to the individual and their families but society, employers, the economy and health service provision. A 2020 analysis reported that mental ill-health costs UK employers up to £45 billion each year, a rise of 16% since 2016 (Deloitte, 2020). On an average, for every £1 spent by employers on workplace mental health interventions, they get £5 back in reduced absenteeism, presenteeism and staff turnover (Deloitte, 2020). The cost of mental health support and services in England totals £34 billion a year (MH Taskforce report, 2016).

Despite continuing growth of the IT workforce and their pivotal role in business productivity, economic and technological development globally, large-scale and longitudinal studies of IT worker mental health are lacking. Specifically, there is an absence of robust research on incident anxiety/depression with comparator groups, consideration of confounding/mediation and that are clinically diagnosed using health records. To the best of our knowledge, no such IT worker studies have been published to date in the UK or globally.

Our study therefore aimed to address this knowledge gap. We evaluated incident diagnosed anxiety/ depression in IT workers compared with the general working population and examined to what extent baseline sociodemographic, health, lifestyle, and occupational factors modify that association. A secondary aim was to evaluate prevalent self-reported mental health outcomes (anxiety/depression symptoms, general practice (GP) or psychiatric attendance for these) and loneliness in IT workers compared with the general working population. For both aims, we repeated these investigations within IT worker subgroups and then comparing IT workers to other similar occupational groups.

## Methods

We conducted a population-based cohort study using UK Biobank data with linkage to GP records.

### Dataset

UK Biobank is a large cohort of over 502 000 participants (5.5% response rate) aged 37–73 years recruited between 2006 and 2010, from across Great Britain. At baseline, participants completed detailed assessments relating to their socio-demographics, lifestyle (including smoking status, alcohol consumption, physical activity, sleep), medical history, physical and mental health on a touchscreen questionnaire and underwent various physical health and biological sample measurements (UK Biobank, 2022).

### Employment status variables and job coding

At recruitment, employment status was recorded for 99% of participants alongside basic occupational exposure data including: tenure in current occupation, working hours, work-related sedentary behaviour and shift work. Those ‘currently employed’ (*n* = 287,137) were interviewed by trained operators to provide job description information to enable spot coding of their job. Using the Standard Occupational Classification (SOC) V.2000, the interviewers coded job titles using a tree structure algorithm. Free-text descriptions that could not be coded by the interviewer (*n* = 18,322) were subsequently SOC-coded using the Computer-assisted Structure Coding Tool (UK Biobank, 2022).

### Study population

**Figure 1:**
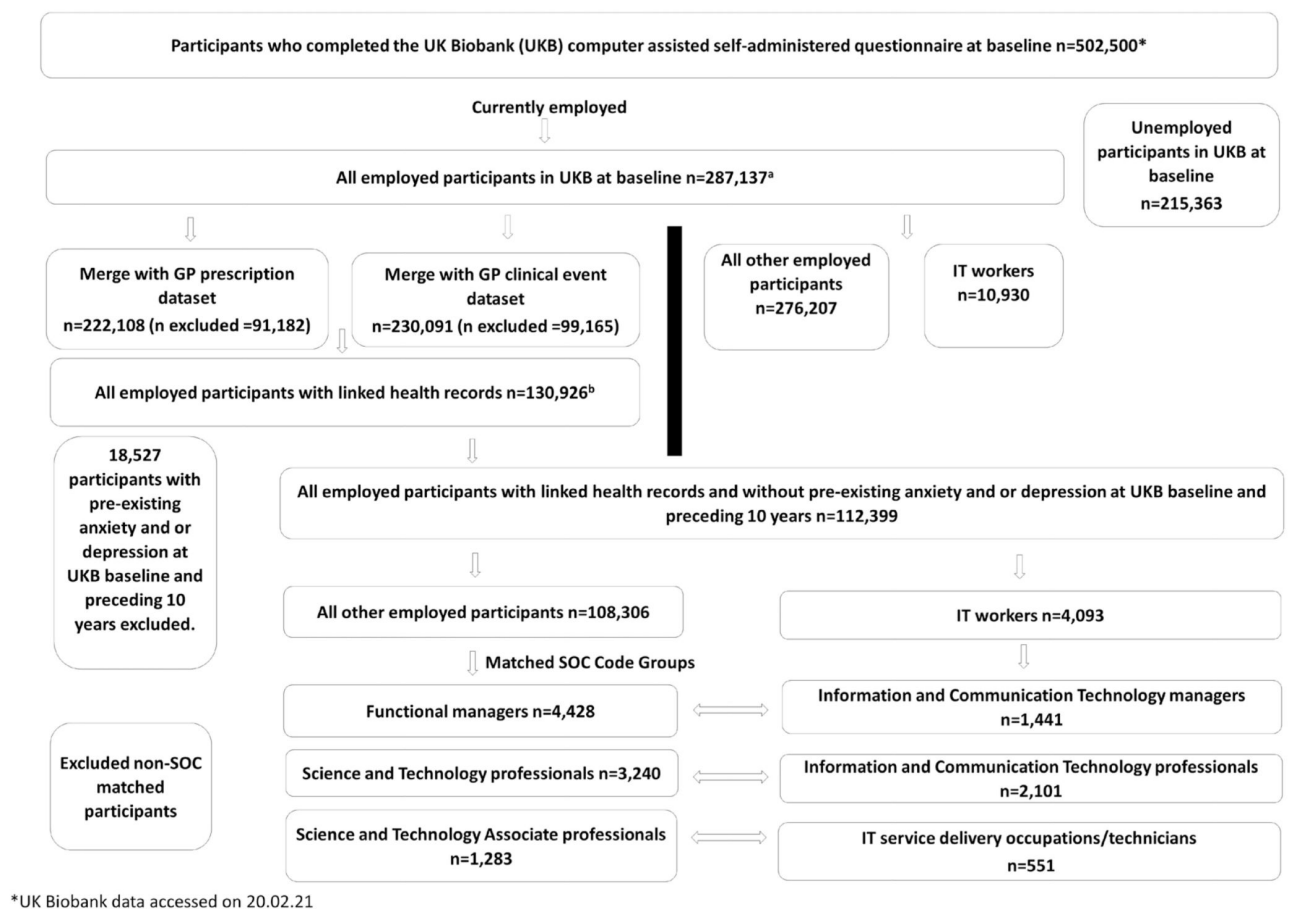
Flow chart of the selection process and samples included in the cross-sectional^a^ and longitudinal^b^ analyses

Our study population comprised IT workers and all other employed Biobank participants (Fig. 1). IT workers were subcategorized into three groups by SOC: IT managers (information and communication technology managers), IT professionals (information and communication technology professionals), and IT technicians (IT service delivery occupations). Comparisons were conducted with their respective counterparts within the same SOC major group of functional managers (FMs), science and technology professionals (STPs), and science and technology associate professionals (STAPs).

### Self-reported mental health variables/outcomes

Anxiety and depression symptoms were measured at baseline via four questions adapted from the Patient Heath Questionnaire-9, for example, “Over the past two weeks, how often have you felt down, depressed or hopeless?” (Martin *et al.*, 2006). See Supplementary Table S1 (available at *Annals of Occupational Hygiene* online) in online edition for full list. Participants selected either “not at all” (scored 0), “several days” (scored 1), “more than half of the days” (scored 2), or “nearly every day” (scored 3). Scores were added to produce a scale from 0 to 12. We applied a previously validated threshold of 0–5 and 6–12 as an indicator of the absence or presence of anxiety/depression symptoms (Löwe *et al.*, 2010).

At baseline, participants were asked questions about loneliness and confiding in others. Similar to the revised UCLA Loneliness Scale and other studies, we combined the questions: “Do you often feel lonely?” (no = 0, yes = 1) and “How often are you able to confide in someone close to you?” (0 = almost daily to once every few months; 1 = never or almost never), to create a total loneliness score (Russell *et al.*, 1980; Elovainio *et al.*, 2017). Participants were defined as lonely if, in total, they scored 2, and not lonely if they scored 0 or 1 (Elovainio *et al.*, 2017).

At baseline, participants were asked if they had: “Ever seen a doctor (GP) for nerves, anxiety, tension or depression” or “Ever seen a psychiatrist for nerves, anxiety, tension, or depression” to which they answered yes or no.

### GP dataset

In 2019, coded primary care/GP data for approximately 45% of the UK Biobank cohort were linked to their records (see Supplementary File in online edition). GP data were available from 1990 to 2016/2017 enabling follow-up of these participants from baseline Biobank recruitment. The GP dataset includes prescription and clinical event records, with corresponding dates.

### Incident GP-diagnosed anxiety/ depression outcome

The primary outcome was defined as a first episode of GP-diagnosed or treated anxiety and/or depression (i.e. first diagnosis (DX+) or first prescription (RX+)). Some incident cases were defined by diagnostic codes without prescription records (DX+, RX-), some were defined by prescription records only (DX-, RX+) and some incident cases were defined by concordant diagnostic and prescription information (DX+, RX+). See Supplementary File in online edition for diagnosis and prescription code lists used and anti-anxiety/depressant selection criteria.

Individuals who died were censored at the time of death and not recorded as having an event. GP data were available for all participants from 1990 until May 2017 for Scotland, September 2017 for Wales and either June/July 2017 or August 2016 for England (month midpoint applied as exact end-dates not provided). End of follow-up was classified as these dates unless preceded by date of death, or the date of a first anxiety/depression diagnosis or prescription.

### Study sample and inclusion criteria

For our primary outcome (incident GP-diagnosed anxiety/depression), we restricted our study sample (Fig. 1) to all employed participants within the UK Biobank at baseline for whom GP records were also available (*n* = 130 926). Participants with pre-existing anxiety/depression (i.e. either an anxiety and/or depression diagnosis or anti-anxiety/depressant prescription) at baseline and the preceding 10 years, were excluded from the analysis (*n* = 18 527).

For our secondary outcomes (self-reported mental health variables), our study sample (Fig. 1) included all employed participants within the UK Biobank at baseline (*n* = 287 137).

### Covariates/potential confounders and mediators

We included potential baseline confounding variables: age, sex, ethnicity, recruitment date; and potential mediators: aggregate-level socioeconomic deprivation (measured using Townsend score) and annual household income. Lifestyle and health factors, i.e. smoking, alcohol consumption, physical activity, sleep duration, body mass index, longstanding illness/disability and work-related factors, i.e. tenure in current occupation, working hours, work-related sedentary behaviour, shift work were also included as potential mediating variables.

### Statistical analysis

Descriptive statistics were used to summarize study population characteristics. Kaplan–Meier plots were used to assess the proportional hazard assumption for comparing IT workers to others in employment, the IT worker subgroups and their SOC counterparts. Having ascertained that the proportional hazards assumption had been met, survival analyses for incident anxiety/depression outcomes were conducted using Cox proportional hazard regression.

Models were applied in a staged process; Model 0 was unadjusted for all covariates; Model 1 adjusted for potential confounders (Model 0 plus age, sex, ethnicity, recruitment date); Model 2 additionally adjusted for potential socio-demographic mediators (Model 1 plus socioeconomic deprivation and annual household income); Model 3 additionally adjusted for potential lifestyle and health mediators (Model 2 plus smoking, alcohol consumption, physical activity, sleep duration, BMI, longstanding illness/disability), and Model 4 further adjusted for potential work-related mediators (Model 3 plus tenure in current occupation, working hours, work-related sedentary behaviour, shift work). These models were repeated for the IT worker subgroups (reference category: managers) and their SOC major group counterparts of FMs, STPs and STAPs, each as the reference categories, respectively.

For the cross-sectional analyses (*n* = 287 137), logistic regression models were used to assess associations between IT worker status (compared to all other employed participants) and self-reported mental health variables (a) Anxiety/depression symptoms, (b) Ever seen a GP for anxiety/depression, (c) Ever seen a psychiatrist for anxiety/depression, and (d) Total loneliness score. The modelling strategy was the same as for the survival analyses. Analyses were performed using statistical software Stata V17 (StataCorp LP).

Multiple imputation by chained equations was used to impute missing data, under a missing at random assumption, creating 20 imputation datasets. All the covariates and outcomes for the Cox proportional hazard and logistic regression models were included in the imputation procedure. All subsequent modelling steps were pooled over the 20 imputation datasets.

## Results

The GP data-linked cohort comprised 112 399 employed participants and 4093 (3.6%) were IT workers (Table 1).

Over three-quarters of IT workers (77%) were male, with a median age of 50 years (25th/75th percentile: 45/55) (Table 1a). IT workers comprised 1441 IT managers, 2101 IT professionals and 551 IT technicians (1.3%, 1.9%, 0.5% of the total employed UK Biobank cohort, respectively; Table 1b). The median age of each subgroup was 50 years (25th/75th percentile: 45/55), 49 years (25th/75th percentile: 44/55) and 51 years (25th/75th percentile: 45/56), respectively, and in all groups the majority were male (75%, 81.1%, 63.3%, respectively). The baseline characteristics of the full Biobank employed population are previously published by Lalloo *et al.* (2021) and their self-reported mental health and psycho-social characteristics are presented in Supplementary Table S1 (available at *Annals of Occupational Hygiene* online).

### Logistic regression analyses

Adjusting for confounders, IT workers overall have a 34% reduced odds of self-reported anxiety/depression symptoms compared to all other employed participants (Model 1: OR = 0.66, 95%CI: 0.52–0.85; Table 2a).

Within the IT worker subgroups, IT technicians have a more than twofold increased odds (Model 1: OR = 2.33, 95%CI: 1.24–4.37) of self-reported anxiety/depression symptoms compared to IT managers (Table 2b).

Adjusting for confounders, within the IT worker subgroups, both IT professionals (Model 1: OR = 1.15, 95%CI: 1.01–1.32) and technicians (Model 1: OR = 1.40, 95%CI: 1.16–1.68) have an increased odds of self-reported loneliness compared to IT managers (Table 2b). Similarly, an increased odds is observed when compared to their SOC counterparts (Model 1: OR = 1.20, 95%CI: 1.07–1.35, Model 1: OR = 1.24, 95%CI: 1.02–1.49 for IT professionals and technicians, respectively) (Table 2c).

Adjusting for confounders, IT workers overall are 13% less likely (Model 1: OR = 0.87, 95%CI: 0.83–0.91) compared to all other employed participants to have ever attended their GP for anxiety/depression (Table 2a). Within the IT worker subgroups, IT technicians are 28% more likely (Model 1: OR = 1.28, 95%CI: 1.11–1.46) compared to IT managers and 22% more likely (Model 1: OR = 1.22, 95%CI: 1.07–1.40) compared to their SOC counterparts to have ever attended their GP for anxiety/depression (Table 2b, 2c).

Within the IT worker subgroups, adjusting for confounders, IT technicians are 32% more likely (Model 1: OR = 1.32, 95%CI: 1.07–1.62) compared to IT managers and 31% more likely (Model 1: OR = 1.31, 95%CI: 1.06–1.62) compared to their SOC counterparts to have ever attended a psychiatrist for anxiety/depression (Table 2b, 2c).

### Survival analyses

The sample size for the incident anxiety/depression outcome in IT workers and all other employed participants was 105 793 participants, with a median follow-up duration of 7.43 years. Adjusting for confounders, IT workers overall have a lower risk (Model 1: HR = 0.84, 95%CI: 0.77–0.93) of incident anxiety/depression compared to all other employed participants (Table 3a).

Within the IT worker subgroups, compared to IT managers, IT professionals (Model 1: HR = 0.92, 95%CI: 0.75–1.12) appear to have a lower risk of incident anxiety/depression and IT technicians a higher risk (Model 1: HR = 1.10, 95%CI: 0.83–1.44), although this did not reach statistical significance (Table 3b). Compared to their respective SOC counterparts, there is a suggestion of an increased risk of incident anxiety/depression for all three IT worker subgroups, i.e. managers (Model 1: HR = 1.04, 95%CI: 0.87–1.23), professionals (Model 1: HR = 1.10, 95%CI: 0.92–1.31) and technicians (Model 1: HR = 1.08, 95%CI: 0.82–1.43), but differences did not reach statistical significance (Table 3c).

Results across successive models: Evaluating our results across the staged models we applied for our outcomes, for both our survival and logistic regression analyses, in a general sense (with the exception of a few), what we hypothesised as mediators seem to be acting that way, with the point estimates in respective models moving towards one. The precision of these estimates varied in terms of statistical significance.

### Additional survival analyses

To take account of chronic, persistent or recurrent cases and potential selection bias, we repeated the survival (complete case) analyses on a population of both the prevalent and incident cases and we applied an adjustment within our models for those who had a pre-existing anxiety/depression diagnosis or prescription (Yes/No). See Supplementary Table S3 (available at *Annals of Occupational Hygiene* online). In this analysis, IT workers have a reduced risk of incident anxiety/depression (Model 1: HR = 0.85, 95%CI: 0.78–0.93) compared with all other employed participants and the findings are similar to our incident only survival (complete case) analyses results—see Supplementary Table S4 (Model 1: HR = 0.84, 95%CI: 0.76–0.93) - and our multiple imputation incident only survival analyses results- see Table 3a (Model 1: HR = 0.84 95%CI 0.77– 0.93). The absence of any material difference when the prevalent cases are included demonstrate that potential selection bias is not likely an issue in this population. To further address potential selection bias, we repeated the incident (complete case) analyses, with stratification on age at baseline (40–55 and 55+ year olds). See Supplementary Table S5 (available at *Annals of Occupational Hygiene* online). IT workers in both the 40–55 (Model 1: HR = 0.86, 95%CI:0.77–0.97) and 55+ (Model 1: HR = 0.76, 95%CI: 0.59–0.98) year groups have a reduced risk of incident anxiety/depression compared with all other employed participants with a lower HR in the 55+ year group.

To address potential outcome heterogeneity, we repeated the survival (complete case) analyses stratifying incident cases into those who had an anxiety/depression diagnosis only and those who had an anxiety/depression prescription only. IT workers have a reduced risk of both an anxiety/depression diagnosis (Model 1: HR = 0.87, 95%CI: 0.77–0.97) and an anxiety/depression prescription (Model 1: HR = 0.78, 95%CI: 0.70–0.88) compared with all other employed participants. See Supplementary Tables S6 & S7 (available at *Annals of Occupational Hygiene* online). However, the HR for IT workers compared with all other employed participants is lower for anxiety/depression prescription than it is for an anxiety/depression diagnosis. If an anxiety/depression prescription is a proxy of severity, then this suggests a potentially lower degree of severity in IT workers, albeit CIs are wide. IT professionals have a reduced risk (Model 1: HR = 0.76, 95%CI: 0.59–0.99) of an anxiety/depression prescription compared to IT managers.

To evaluate potential measurement error in exposure, we repeated the incident analyses, with stratification on duration of employment (<10 years and ≥10 years) in IT at baseline. See Supplementary Table S8 (available at *Annals of Occupational Hygiene* online). IT workers in both the < 10 (Model 1: HR = 0.85, 95%CI: 0.74–0.98) and ≥ 10 (Model 1: HR = 0.84, 95%CI: 0.72–0.98) years of employment groups have a similarly reduced risk of incident anxiety/depression compared with all other employed participants with very similar HRs.

## Discussion

In this study, we observed that overall, compared to all other employed participants, IT workers had a lower risk of incident anxiety/depression, a 34% reduced odds of anxiety/depression symptoms and are 13% less likely to have ever attended their GP for anxiety/depression. Further, we found that loneliness is greater in IT professionals and technicians when compared to IT managers, and their respective SOC groups.

IT technicians have an increased odds of anxiety/ depression symptoms compared to IT managers and are more likely, compared to IT managers and their respective SOC group, to present with anxiety/depression requiring GP care and specialist psychiatric input. The latter suggests higher disease severity in this group, as clinically, only severe or intractable cases are escalated for psychiatric input.

There is a suggestion from our survival analyses, of potential adverse mental health outcomes in the IT worker subgroup and SOC group comparisons, but the differences did not reach statistical significance. This could be due to the relatively small sample size in this dataset, compared with the larger cross-sectional dataset.

There is a paucity of formal research on diagnosed or treated mental health conditions in IT workers. Existing studies focus on increased exposure risks to work-related stress and burnout (Fujigaki *et al.*, 1994; Sonnentag *et al.*, 1994; Salanova *et al.*, 2002; Kouvonen *et al.*, 2005; Sharma *et al.*, 2006; Pawlowski *et al.*, 2007; Rashidi and Jalbani, 2009; Darshan *et al.*, 2013; Shih *et al.*, 2013). In a BIMA (2019) member survey of the UK digital and technology community, 52% reported they had suffered from anxiety/depression; 28% reported a mental health diagnosis. A handful of other studies include self-reported anxiety/depression symptoms but as with the BIMA survey, they are all cross-sectional without consideration of confounding or mediation and only one had a comparator group (Rocha and Debert-Ribeiro, 2004; Vimala and Madhavi, 2009; Nayak, 2014; Padma *et al.*, 2015).

Our main finding of a reduced risk of diagnosed or treated anxiety/depression in IT workers compared to the general employed population is novel, and somewhat divergent from the self-reporting cross-sectional IT worker studies above (that observed increased anxiety and depression symptoms), notably the BIMA survey which reported that digital and technology workers are five times more likely to have suffered anxiety or depression compared to general population estimates. However, the proportion of IT workers in that survey is unclear, their respondents were younger (78% were under 45 years) than our IT worker population (over 40s only) and, in the other studies, these were of IT workers undertaking task-specific roles (Rocha and Debert-Ribeiro, 2004; Nayak, 2014).

One potential explanation for our findings could be the older age demographic of our IT worker population, with more experience and adaptation to their roles over time. This is supported by our age stratification analyses with a lower HR in the older age group. Another explanation may be that, although IT work is mentally intense, rapidly changing and demanding, it is also highly innovative, creative and challenging. Job satisfaction and self-selection of individuals who thrive on being challenged and are better able to cope with change and pressure, could be a mitigating factor to the development of stress-related mental illness. This hypothesis is also described by Ivancevich *et al.* (1983).

Regarding our findings on self-reported loneliness in IT workers, lower social support among IT managers and work-family conflict in the IT workforce have been reported (Weiss, 1983; Rocha and Debert-Ribeiro, 2004; Hoonakker *et al.*, 2005). The latter has been associated with the IT worker’s fascination by the computer/ technology, ‘IT dependency’ and replacement of human interaction, and high work participation levels (Rocha and Debert-Ribeiro, 2004; Pawlowski *et al.*, 2007). IT work organisation and remote working can result in colleague disengagement and isolation (BIMA, 2019). The proportion of neurodivergent individuals are reportedly twice as high in the IT industry than the general population, and while these individuals naturally self-selected into IT work initially, IT companies are now actively recruiting neurodiverse talent for their specific traits (BIMA, 2019). While these individuals may thrive in their work, socially they may struggle, with potential isolation/loneliness.

While existing literature has focussed on the psychological health of more skilled IT workers particularly, software professionals and systems analysts, studies investigating comparatively lower-skilled groups, such as IT technicians are lacking (Rocha and Debert-Ribeiro, 2004; Nayak, 2014). Less innovation/creativity, lower reward, and a more demanding customer-facing role, e.g. helpdesk support, may be explanations for the higher mental health attendances of IT technicians observed in our study.

This novel UK-based study is the largest to date examining IT worker mental health, with a rich characterisation of variables. It is the first longitudinal incidence study of IT worker mental health with comparator groups (both general employed population and similar occupations) and with consideration of confounding and mediation. It is also the first to evaluate diagnosed or treated anxiety/depression in IT workers using health records. Our study is not restricted to a single IT company or sector but covers those working across a range of organizations/sectors, providing a broader, more generalizable picture of the mental health risks of IT work. Given our study population age demographics (40+ years), those older participants with life-long IT careers are likely to represent the ‘pioneering’ generation of UK IT workers who have worked through the entire journey of the IT revolution (from inception), with the longest exposure history to this type of work to date. This unique feature gives us important insight into the longer-term exposure to IT work and related mental health trajectories.

UK Biobank’s low response rates, selection bias due to the recruitment criteria and healthy-worker effect are potential limitations but there is debate that despite this, risk factor associations in the UK Biobank seem to be generalizable (Munafò *et al.*, 2017; Batty *et al.*, 2020). Recruitment ceasing in 2010 with no subsequent update to the occupational status of our study participants is also a limitation. The proportionally lower number of IT technicians (compared to IT professionals and IT managers) reduces power to make inferences. The absence in UK Biobank of subjects aged less than 40, means our results may not generalize to younger IT workers. We accounted for socioeconomic factors in our models, but residual confounding remains possible. With the rapid evolution of IT over the past decade, it is possible that the nature of IT work and working conditions have changed and our results may not be reflective of these changes. Furthermore, there is well-established recognition that a substantial burden of common mental health disorders do not present for clinical treatment and a substantial proportion of patients presenting for the treatment of mental health symptoms have mild illness.

Further research in other cohorts and settings is advocated to replicate/validate our findings. The increased loneliness also merits further research to identify clear explanations and consequently, appropriate workplace social support strategies to mitigate this.

## Conclusions

Our findings set a benchmark in our understanding of IT worker mental health and are a stepping stone to identifying high-risk groups and psychosocial factors, which will have important implications for targeting and informing workplace practices and mental health workplace interventions. This will assist in improving IT worker mental health and increasing the work participa-tion and retention of this under-researched and rapidly growing occupational group, on whom our daily and working lives, businesses and the global economy have become so highly dependent.

## Supplementary Material

Supplementary Material

## Figures and Tables

**Table 1:** Socio-demographic, health, lifestyle and work characteristics in (a) the IT worker group compared to all other employed Biobank participants and (b) within IT worker subgroups. Longitudinal study population: all employed Biobank participants with linked GP records

	a		b		
	All other employed Biobank participants	All IT workers	IT managers	IT professionals	IT technicians
Total *n* (%)112 399 (100)	108 306 (96.4)	4093 (3.6)	1441 (1.3)	2101 (1.9)	551 (0.5)
**Socio-demographic factors**					
Sex					
Male	53 388 (49.3)	3133 (76.6)	1081 (75)	1703 (81.1)	349 (63.3)
Female	54 918 (50.7)	960 (23.5)	360 (25)	398 (19)	202 (36.7)
Age (years), median (IQR;Q1/Q3)	53 (12; 47/59)	50 (10; 45/55)	50 (10; 45/55)	49 (11; 44/55)	51 (11; 45/56)
Age (years)					
40–44*	16 525 (15.3)	1016 (24.8)	342 (23.7)	549 (26.1)	125 (22.7)
45–49	21 479 (19.8)	1031 (25.2)	378 (26.2)	532 (25.3)	121 (22)
50–54	23 928 (22.1)	944 (23.1)	350 (24.3)	458 (21.8)	136 (24.7)
55–59	24 299 (22.4)	678 (16.6)	249 (17.3)	328 (15.6)	101 (18.3)
60–64	17 540 (16.2)	367 (9)	108 (7.5)	203 (9.7)	56 (10.2)
65+	4535 (4.2)	57 (1.4)	14 (1)	31 (1.5)	12 (2.2)
Ethnicity					
White	102 290 (94.5)	3850 (94.1)	1374 (95.4)	1954 (93)	522 (94.7)
Asian	2417 (2.2)	120 (2.9)	35 (2.4)	74 (3.5)	11 (2)
Missing**	3599 (3.3)	123 (3)	32 (2.2)	73 (3.5)	18 (3.3)
Townsend deprivation index					
1 (least deprived quintile)	48 866 (45.1)	2079 (50.8)	829 (57.5)	1034 (49.2)	216 (39.2)
2	24 979 (23.1)	924 (22.6)	304 (21.1)	480 (22.9)	140 (25.4)
3	17 115 (15.8)	612 (15)	186 (12.9)	333 (15.9)	93 (16.9)
4	12 647 (11.7)	374 (9.1)	91 (6.3)	204 (9.7)	79 (14.3)
5 (most deprived quintile)	4515 (4.2)	98 (2.4)	30 (2.1)	47 (2.2)	21 (3.8)
Missing**	184 (0.2)	6 (0.2)	1 (0.1)	3 (0.1)	2 (0.4)
Household Annual Income (£)					
Less than £18 000	10 437 (9.6)	83 (2.0)	16 (1.1)	44 (2.1)	23 (4.2)
£18 000 to £30 999	22 369 (20.7)	373 (9.1)	60 (4.2)	193 (9.2)	120 (21.8)
£31 000 to £51 999	31 283 (28.9)	1318 (32.2)	368 (25.5)	741 (35.3)	209 (37.9)
£52 000 to £100 000	26 928 (24.9)	1708 (41.7)	721 (50)	856 (40.7)	131 (23.8)
Greater than £100 000	6979 (6.4)	369 (9.0)	199 (13.8)	153 (7.3)	17 (3.1)
Missing**	10 310 (9.5)	242 (5.9)	77 (5.3)	114 (5.4)	51 (9.3)
**Health and lifestyle factors**					
Long standing illness, disability or infirmity					
Yes	25 931 (23.9)	945 (23.1)	324 (22.5)	483 (23)	138 (25.1)
No	80 140 (74)	3093 (75.6)	1099 (76.3)	1587 (75.5)	407 (73.9)
Missing**	2235 (2.1)	55 (1.3)	18 (1.3)	31 (1.5)	6 (1.1)
Body mass index (kg/m^2^)					
<25	36 418 (33.6)	1337 (32.7)	446 (31)	725 (34.5)	166 (30.1)
≥25	71 465 (66)	2747 (67.1)	994 (69)	1370 (65.2)	383 (69.5)
Missing**	423 (0.4)	9 (0.2)	1 (0.1)	6 (0.3)	2 (0.4)
Smoking status					
Never smoker	62 965 (58.1)	2611 (63.8)	885 (61.4)	1372 (65.3)	354 (64.3)
Previous/Current smoker	45 059 (41.6)	1474 (36)	553 (38.4)	725 (34.5)	196 (35.6)
Missing**	282 (0.3)	8 (0.2)	3 (0.2)	4 (0.2)	1 (0.2)
Alcohol consumption+(units/week)					
≤14	22 046 (20.4)	824 (20.1)	290 (20.1)	411 (19.6)	123 (22.3)
>14	55 238 (51)	2356 (57.6)	871 (60.4)	1221 (58.1)	264 (47.9)
Missing**	31 022 (28.6)	913 (22.3)	280 (19.4)	469 (22.3)	164 (29.8)
Physical activity (MET min/week) <600	15 367 (14.2)	641 (15.7)	233 (16.2)	322 (15.3)	86 (15.6)
≥600	49 477 (45.7)	1830 (44.7)	635 (44.1)	950 (45.2)	245 (44.5)
Missing**	43 462 (40.1)	1622 (39.6)	573 (39.8)	829 (39.5)	220 (39.9)
Sleep (h/day)					
≥7	80 779 (74.6)	3076 (75.2)	1070 (74.3)	1590 (75.7)	416 (75.5)
<7	27 145 (25.1)	1015 (24.8)	370 (25.7)	510 (24.3)	135 (24.5)
Missing**	382 (0.4)	2 (0.1)	1 (0.1)	1 (0.1)	0 (0)
**Occupational factors**					
Tenure in current job (not employer), years					
<1	5320 (4.9)	201 (4.9)	64 (4.4)	121 (5.8)	16 (2.9)
1–5	28 980 (26.8)	1012 (24.8)	353 (24.5)	531 (25.3)	128 (23.2)
6–10	22 163 (20.5)	927 (22.7)	289 (20.1)	517 (24.6)	121 (22.)
11–15	13 194 (12.2)	582 (14.2)	182 (12.6)	329 (15.7)	71 (12.9)
16–20	12 463 (11.5)	451 (11)	169 (11.7)	208 (9.9)	74 (13.4)
21–25	9079 (8.4)	399 (9.8)	144 (10)	192 (9.1)	63 (11.4)
Missing	17 107 (15.8)	521 (12.7)	240 (16.7)	203 (9.7)	78 (14.2)
Working hours per week					
≤38	57 515 (53.1)	1861 (45.5)	469 (32.6)	1065 (50.7)	327 (59.4)
>38	49 210 (45.4)	2208 (54)	965 (67)	1021 (48.6)	222 (40.3)
Missing**	1581 (1.5)	24 (0.6)	7 (0.5)	15 (0.7)	2 (0.4)
Job involves walking/standing					
Always/Usually/Sometimes	72 527 (67)	1086 (26.5)	407 (28.2)	450 (21.4)	229 (41.6)
Never/Rarely	35 630 (32.9)	3007 (73.5)	1034 (71.8)	1651 (78.6)	322 (58.4)
Missing**	149 (0.14)	0 (0)	0 (0)	0 (0)	0 (0)
Job involves shift work					
Always/Usually/Sometimes	19 441 (18)	292 (7.1)	67 (4.7)	150 (7.1)	75 (13.6)
Never/Rarely	88 599 (81.8)	3798 (92.8)	1373 (95.3)	1950 (92.8)	475 (86.2)
Missing**	266 (0.3)	3 (0.1)	1 (0.1)	1 (0.1)	1 (0.2)

Column a= Comparison of all IT workers and all other employed Biobank participants.

Column b= Comparison of IT worker subgroups: IT managers, IT professionals and IT technicians.

*N* = number; MET = metabolic equivalent.

* 35–39 year olds added to this total due to very small numbers *n* = 2.

** includes ‘missing’, ‘do not know’ and ‘prefer not to answer’ responses.

+ The recommended alcohol consumption guidelines changed in 2016 (i.e. following baseline data collection) from 21 units/week for women and 28 units/week for men to these current thresholds of 14 units/week for men and women.

**Table 2:** Logistic regression models” in (a) all IT workers compared to all other employed Biobank participants, (b) IT worker subgroups (managers, professionals, technicians) and (c) IT subgroups compared to other similar occupations within their SOC tree (Functional Managers, Science and Technology Professionals, Science and Technology Associate Professionals) for self-reported mental health outcomes and loneliness. Cross-sectional study population: all employed Biobank participants at baseline

	a		b				c					
	All IT workers compared to all other employed Biobank participants		IT worker subgroups				IT worker subgroups compared to other similar occupations within their SOC tree.					
	(Reference Category: *All other employed Biobank participants*)		(Reference Category: *IT managers*)				(Reference Category: *All other Functional managers*¥)		(Reference Category: *All other Science & Technology professionals*¥)		(Reference Category: *All other Science & Technology Associate professionals*¥)	
Total я (%) 287137 (100)	All IT workers		IT professionals		IT technicians		IT managers		IT professionals		IT technicians	
	276 207(96.2)		10 930 (3.8)				3698 (1.3)		5755 (2.0)		1477(0.5)	
	OR	95% CI	OR	95% CI	OR	95% CI	OR	95% CI	OR	95% CI	OR	95% CI
**Anxiety/depression symptoms** x												
*(No symptoms*¥)												
Model 0^d^	0.65	0.51–0.83	0.95	0.53–1.68	2.4	1.28–4.50	0.89	0.54–1.47	0.91	0.57–1.46	1.62	0.89–2.94
Model 1^e^	0.66	0.52–0.85	0.95	0.54–1.69	2.33	1.24–4.37	0.9	0.54–1.47	0.82	0.51–1.31	1.46	0.80–2.65
Model 2^f^	0.89	0,70–1.14	0.82	0.46–1.45	1.61	0.85–3.02	0.93	0.57–1.53	0.84	0.52–1.34	1.56	0.86–2.83
Model 2^g^	0.89	0.70–1.14	0.84	0.47–1.49	1.58	0.84–2.98	0.91	0.55–1.50	0.82	0.51–1.31	1.51	0.83–2.74
Model 4^h^	0.92	0.72–1.17	0.83	0.47–1.47	1.61	0.86–3.03	0.91	0.55–1.50	0.84	0.52–1.34	1.51	0.83–2.75
**Loneliness score** ¥												
*(Not lonely*¥)												
Model 0^d^	0.82	0.77–0.87	1.12	0.98–1.28	1.52	1.27–1.83	0.95	0.84–1.07	1.28	1.14–1.44	1.34	1.11–1.61
Model 1^e^	0.96	0.90–1.02	1.15	1.01–1.32	1.4	1.16–1.68	1.02	0.91–1.16	1.2	1.07–1.35	1.24	1.02–1.49
Model 2^f^	1.15	1.08–1.22	1.05	0.91–1.20	1.1	0.92–1.33	1.05	0.93–1.19	1.21	1.08–1.35	1.28	1.06–1.55
Model 3^g^	1.15	1.08–1.22	1.06	0.92–1.22	1.1	0.91–1.32	1.04	0.92–1.18	1.2	1.07–1.35	1.26	1.05–1.53
Model 4^h^	1.14	1.07–1.22	1.06	0.92–1.22	1.1	0.92–1.33	1.05	0.93–1.19	1.18	1.05–1.33	1.24	1.03–1.50
**Ever seen a GP for nerves, anxiety, tension or depression**												
*(No*¥)												
Model 0^d^	0.7	0.67–0.73	0.94	0.85–1.04	1.41	1.23–1.61	0.88	0.80–0.95	1.08	0.99–1.17	1.29	1.13–1.47
Model 1^e^	0.87	0.83–0.91	0.99	0.90–1.09	1.28	1.11–1.46	0.98	0.90–1.07	1.04	0.96–1.13	1.22	1.07–1.40
Model 2^f^	0.95	0.91–0.99	0.93	0.84–1.02	1.11	0.96–1.27	0.98	0.90–1.07	1.04	0.96–1.13	1.24	1.08–1.43
Model 3^g^	0.96	0.92–1.00	0.94	0.85–1.04	1.09	0.95–1.25	0.99	0.91–1.08	1.03	0.94–1.11	1.21	1.06–1.40
Model 4^h^	0.93	0.89–0.97	0.9	0.82–0.99	1.07	0.93–1.23	1	0.91–1.09	0.96	0.89–1.05	1.18	1.03–1.36
Ever seen a psychiatrist for nerves, anxiety, tension or depression												
*(No*¥)												
Model 0^d^	0.86	0.80–0.92	1.1	0.95–1.29	1.37	1.12–1.69	0.91	0.80–1.05	1.14	1.01–1.29	1.34	1.08–1.65
Model 1^e^	0.93	0.87–1.00	1.12	0.97–1.31	1.32	1.07–1.62	0.95	0.83–1.09	1.13	1.00–1.28	1.31	1.06–1.62
Model 2^f^	1.04	0.97–1.12	1.06	0.91–1.24	1.15	0.94–1.42	0.97	0.84–1.11	1.12	0.99–1.26	1.33	1.08–1.65
Model 3^g^	1.05	0.98–1.13	1.07	0.92–1.25	1.13	0.92–1.39	0.97	0.85–1.11	1.1	0.97–1.25	1.3	1.05–1.61
Model 4^h^	1.02	0.95–1.10	1.03	0.88–1.20	1.11	0.90–1.37	0.98	0.85–1.12	1.03	0.91–1.17	1.26	1.02–1.56

x Anxiety/depression symptoms score (0-12): Absence of anxiety or depression symptoms (Total score = 0-5), Presence of anxiety or depression symptoms (Total score = 6-12).

¥ Loneliness score (0-2): Not lonely (Total score = 0,1), Lonely (Total score = 2).

*Italics* and ¥ denote the reference category.

OR, odds ratio; CI, confidence interval.

Model 0^d^ = Unadjusted.

Model 1^e^ = Model 0 + sociodemographic factors i.e. confounders (age, sex, ethnicity, date of Biobank recruitment/baseline assessment).

Model 2^f^ = Model 1 + additional sociodemographic factors i.e. potential mediators (deprivation, annual household income).

Model 3^g^ = Model 2 + health and lifestyle factors (smoking, alcohol, physical activity, sleep duration, BMI, longstanding illness/disability).

Model 4^h^ = Model 3 + work-related factors (tenure in current occupation, working hours, work-related sedentary behaviour, shift work).

**Table 3:** Cox proportional hazard models of the association between socio-demographic, health, lifestyle and work characteristics and incident anxiety/depression in (a) all IT workers compared to all other employed Biobank participants, (b) IT worker subgroups (managers, professionals, technicians) and (c) IT subgroups compared to other similar occupations within their SOC tree (Functional Managers, Science and Technology Professionals, Science and Technology Associate Professionals). Longitudinal study population: all employed Biobank participants with linked GP records

		Model 0^d^	Model 1^e^	Model 2^f^	Model 3^g^	Model 4^h^
		Unadjusted HR (95% CI)	Adjusted HR (95% CI)	Adjusted HR (95% CI)	Adjusted HR (95% CI)	Adjusted HR (95% CI)
**A**	Failures 16 351 All other employed participants Incidence rate* (2.2)	1.00	1.00	1.00	1.00	1.00
	All IT workers Incidence rate* (1.7)	0.77 (0.70–0.84)	0.84 (0.77–0.93)	0.93 (0.84–1.01)	0.93 (0.85–1.02)	0.97 (0.89–1.07)
**B**	Failures 477 IT managers Incidence rate* (1.7)	1.00	1.00	1.00	1.00	1.00
	IT professionals Incidence rate* (1.5)	0.88 (0.72–1.07)	0.92 (0.75–1.12)	0.86 (0.70–1.05)	0.87 (0.71–1.07)	0.86 (0.70–1.06)
	IT technicians Incidence rate* (2.0)	1.13 (0.87–1.49)	1.10 (0.83–1.44)	0.93 (0.70–1.23)	0.93 (0.70–1.24)	0.88 (0.66–1.18)
**C**	Failures 712 All other Functional managers Incidence rate* (1.8)	1.00	1.00	1.00	1.00	1.00
	IT managers Incidence rate* (1.7)	0.98 (0.82–1.16)	1.04 (0.87–1.23)	1.03 (0.87–1.22)	1.03 (0.86–1.22)	1.08 (0.91–1.29)
	Failures 526 All other Science & Technology professionals Incidence rate* (1.3)	1.00	1.00	1.00	1.00	1.00
	IT professionals Incidence rate* (1.5)	1.19 (1.00–1.41)	1.10 (0.92–1.31)	1.10 (0.92–1.31)	1.09 (0.91–1.30)	1.12 (0.92–1.37)
	Failures 228 All other Science & Technology Associate professionals Incidence rate* (1.7)	1.00	1.00	1.00	1.00	1.00
	Incidence rate* (2.0)	1.16 (0.88–1.53)	1.08 (0.82–1.43)	1.07 (0.81–1.43)	1.07 (0.81–1.42)	1.11 (0.82–1.51)

HR, hazard ratio; CI, confidence interval.

Model 0^d^ = Unadjusted.

Model 1^e^ = Model 0 + sociodemographic factors i.e. confounders (age, sex, ethnicity, date of Biobank recruitment/baseline assessment).

Model 2^f^ = Model 1 + additional sociodemographic factors i.e. potential mediators (deprivation, annual household income).

Model 3^g^ = Model 2 + health and lifestyle factors (smoking, alcohol, physical activity, sleep duration, BMI, longstanding illness/disability).

Model 4^h^ = Model 3 + work-related factors (tenure in current occupation, working hours, work-related sedentary behaviour, shift work).

* Rates are expressed per 100 and based on person-years.

## Data Availability

Data may be obtained from a third party and are not publicly available.

## What’s Important About This Paper?

In this, the first longitudinal study of IT worker mental health and the first to examine incident mental health conditions, IT workers have a lower overall risk of incident anxiety/depression compared to all other employed UK Biobank participants. Compared to IT managers and workers with similar occupational classifications, IT technicians have a higher odds of anxiety/depression requiring GP or psychiatric attendance. Self-reported loneliness is higher in IT professionals and technicians compared to IT managers and workers with similar occupational classifications. This study sets a benchmark in our understanding of IT worker mental health and for large-scale IT worker mental health studies. It identifies high-risk groups and psychosocial factors which can have important implications for targeting and informing effective mental health workplace interventions.
