# Supplementary material for: Comparing Anxiety and Depression in Information Technology Workers with Others in Employment: A UK Biobank Cohort Study
Source: Ann Work Expo Health. Author manuscript; Available in PMC 2022 Nov 17. (PMC9664232; doi:10.1093/annweh/wxac061)
Supplement: Supplementary Material [file EMS153634-supplement-Supplementary_Material.pdf]

Title: Comparing anxiety and depression in Information Technology workers with others in employment: A UK Biobank cohort study

Author(s): Drushca Lalloo, Jim Lewsey, Srinivasa Vittal Katikireddi, Ewan B Macdonald, Desmond Campbell, Evangelia Demou.

SUPPLEMENTARY MATERIAL

Supplementary Table S1. Self-reported mental health and psychosocial characteristics in a) all IT workers compared to all other employed Biobank participants b) IT worker subgroups (managers, professionals, technicians) and c) IT subgroups compared to other similar occupations within their SOC tree (Functional Managers, Science and Technology Professionals, Science and Technology Associate Professionals)

Cross-sectional study population: all employed biobank participants at baseline

	a		b			c		
	**All other employed Biobank participants**	**All IT workers**	**IT managers**	**IT professionals**	**IT technicians**	**All other Functional managers**	**All other Science &Technology professionals**	**All other Science & Technology Associate professionals**
Total n (%) 287,137 (100)	276,207 (96.2)	10,930 (3.8)	3,698 (1.3)	5,755 (2.0)	1,477 (0.5)	11,767 (4.1)	8,158 (2.8)	3,201 (1.1)
**Self-reported mental ill-health**								
Ever seen a GP for nerves, anxiety, tension or depression								
No	185,453 (67.1)	8,158 (74.6)	2,772 (75)	4,380 (76.1)	1,006 (68.1)	8,520 (72.4)	6,315 (77.4)	2,342 (73.2)
Yes	88,897 (32.2)	2,732 (25)	913 (24.7)	1,353 (23.5)	466 (31.6)	3,203 ((27.2)	1,812 (22.2)	841 (26.3)
Missing	1,857 (0.7)	40 (0.4)	13 (0.4)	22 (0.4)	5 (0.3)	44 (0.4)	31 (0.4)	18 (0.5)
Ever seen a psychiatrist for nerves, anxiety, tension or depression								
No	248,115 (89.8)	9,967 (91.2)	3,403 (92)	5,248 (91.2)	1,316 (89.1)	10,751 (91.4)	7,518 (92.2)	2,936 (91.7)
Yes	26,966 (9.8)	930 (8.5)	288 (7.8)	489 (8.5)	153 (10.4)	998 (8.5)	616 (7.6)	254 (7.9)
Missing	1,126 (0.4)	33 (0.3)	7 (0.2)	18 (0.3)	8 (0.5)	18 (0.1)	24 (0.2)	11 (0.3)
**Self-reported psychological distress:** PHQ (4 items)								
Frequency of depressed mood in last 2 weeks								
Not at all	200,838 (72.7)	8,288 (75.8)	2,861 (77.4)	4,355 (75.7)	1,072 (72.6)	9,134 (77.6)	6,417 (78.7)	2,382 (74.4)
Several days	51,681 (18.7)	2,054 (18.8)	674 (18.2)	1,092 (19)	288 (19.5)	2,037 (17.3)	1,313 (16.1)	578 (18.1)
More than half the days	7,694 (2.8)	238 (2.2)	64 (1.7)	136 (2.4)	38 (2.6)	223 (1.9)	143 (1.8)	65 (2.0)
Nearly every day	4,131 (1.5)	124 (1.1)	43 (1.2)	58 (1)	23 (1.6)	113 (1.0)	63 (0.8)	43 (1.3)
Missing	11,863 (4.3)	226 (2.1)	56 (1.5)	114 (2)	56 (3.8)	260 (2.2)	222 (2.7)	133 (4.2)
Frequency of unenthusiasm/ disinterest in last 2 weeks								
Not at all	208,705 (75.6)	8,318 (76.1)	2,863 (77.4)	4,358 (75.7)	1,098 (74.3)	9,400 (79.9)	6,435 (78.9)	2,415 (75.5)
Several days	45,532 (16.5)	2,026 (18.5)	658 (17.8)	1,097 (19.1)	271 (18.4)	1,802 (15.3)	1,301 (16)	565 (17.7)
More than half the days	7,421 (2.7)	218 (2)	65 (1.8)	114 (2)	39 (2.6)	203 (1.7)	129 (1.6)	86 (2.7)

Nearly every day	4,839 (1.8)	163 (1.5)	43 (1.2)	88 (1.5)	32 (2.2)	151 (1.3)	99 (1.2)	43 (1.3)
Missing	9,710 (3.5)	204 (1.9)	69 (1.9)	98 (1.7)	37 (2.5)	211 (1.8)	194 (2.4)	92 (2.9)
Frequency of tenseness/ restlessness in last 2 weeks								
Not at all	193,457 (70)	7,801 (71.4)	2,613 (70.7)	4,172 (72.5)	1,016 (68.8)	8,339 (70.9)	6,069 (74.4)	2,251 (70.3)
Several days	61,192 (22.2)	2,573 (23.5)	898 (24.3)	1,322 (23)	353 (23.9)	2,854 (24.3)	1,682 (20.6)	730 (22.8)
More than half the days	7,183 (2.6)	199 (1.8)	72 (2)	94 (1.6)	33 (2.2)	210 (1.8)	148 (1.8)	68 (2.1)
Nearly every day	4,162 (1.5)	137 (1.3)	52 (1.4)	61 (1.1)	24 (1.6)	150 (1.3)	82 (1.0)	47 (1.5)
Missing	10,213 (3.7)	220 (2)	63 (1.7)	106 (1.8)	51 (3.5)	214 (1.8)	177 (2.2)	105 (3.3)
Frequency of tiredness/lethargy in last 2 weeks								
Not at all	118,402 (42.9)	5,139 (47)	1,740 (47.1)	2,762 (48)	637 (43.1)	5,511 (46.8)	4,082 (50.0)	1,432 (44.7)
Several days	116,578 (42.2)	4,533 (41.5)	1,551 (41.9)	2,372 (41.2)	610 (41.3)	5,004 (42.5)	3,249 (39.8)	1,340 (41.9)
More than half the days	17,941 (6.5)	638 (5.8)	217 (5.9)	317 (5.5)	104 (7)	626 (5.3)	379 (4.7)	188 (5.9)
Nearly every day	15,735 (5.7)	479 (4.4)	158 (4.3)	230 (4)	91 (6.2)	451 (3.8)	295 (3.6)	157 (4.9)
Missing	7,551 (2.7)	141 (1.3)	32 (0.9)	74 (1.3)	35 (2.4)	175 (1.5)	153 (1.9)	84 (2.6)
**Anxiety/depression symptoms PHQ total score (0-12)**								
0-5 (No symptoms)	247,493 (89.6)	10,304 (94.3)	3,523 (95.3)	5,446 (94.6)	1,335 (90.4)	11,081 (94.2)	7,580 (92.9)	2,899 (90.6)
6-12 (Symptoms)	2,421 (0.9)	68 (0.6)	20 (0.5)	29 (0.5)	19 (1.3)	70 (0.6)	44 (0.5)	24 (0.8)
Missing	26,293 (9.5)	558 (5.1)	155 (4.2)	280 (4.5)	123 (8.3)	616 (5.2)	534 (6.6)	278 (8.6)
**UCLA Loneliness Scale (2 questions below)**								
Do you often feel lonely?								
No	223,180 (80.8)	9,065 (82.9)	3,155 (85.3)	4,738 (82.3)	1,172 (79.4)	10,038 (85.3)	7,027 (86.1)	2,637 (82.4)
Yes	48,536 (17.6)	1,741 (15.9)	508 (13.7)	948 (16.5)	285 (19.3)	1,632 (13.9)	1,025 (12.6)	516 (16.1)
Missing	4,491 (1.6)	124 (1.1)	35 (1)	69 (1.2)	20 (1.4)	97 (0.8)	106 (1.3)	48 (1.5)
How often are you able to confide in someone close to you?								
Almost daily to once every few months	231,990 (84)	8,954 (81.9)	3,088 (83.5)	4,654 (80.9)	1,212 (82.1)	10,154 (86.3)	6619 (81.1)	2543 (79.4)
Never or almost never	36,200 (13.1)	1,730 (15.8)	563 (15.2)	946 (16.4)	221 (15)	1,423 (12.1)	1,327 (16.3)	549 (17.2)
Missing	8,017 (2.9)	246 (2.3)	47 (1.3)	155 (2.7)	44 (3)	190 (1.6)	212 (2.6)	109 (3.4)
**UCLA Loneliness Scale Total score (0-2)**								
0-1 (Not lonely)	228,650 (82.8)	9,372 (85.8)	3,252 (87.9)	4,913 (85.4)	1,207 (81.7)	10,267 (87.3)	7,153 (87.7)	2,699 (84.3)
2 (Lonely)	35,670 (12.9)	1,199 (11)	366 (9.9)	623 (10.8)	210 (14.2)	1,222 (10.4)	701 (8.6)	347 (10.8)
Missing	11,887 (4.3)	359 (3.3)	80 (2.2)	219 (3.8)	60 (4.1)	278 (2.3)	304 (3.7)	155 (4.8)
**Risk taking** Would you describe yourself as someone who takes risks?								
No	185,911 (67.3)	7,282 (66.6)	2,266 (61.3)	3,884 (67.5)	1,132 (76.6)	6,840 (58.1)	5,348 (65.6)	2,335 (73.0)
Yes	79,963 (29)	3,335 (30.5)	1,354 (36.6)	1,685 (29.3)	296 (20)	4,635 (39.4)	2,553 (31.3)	744 (23.2)
Missing	10,333 (3.7)	313 (2.9)	78 (2.1)	186 (3.2)	49 (3.3)	292 (2.5)	257 (3.2)	122 (3.8)

UK Biobank linkage with GP dataset

In 2019, coded primary care/GP data for approximately 45% of the UK Biobank cohort were linked to their records. This linkage was based on having a compatible GP system namely: EMIS and Vision (for Scotland and Wales) and TPP and Vision (for England). The GP dataset includes prescription and clinical event records, with corresponding dates. See https://biobank.ctsu.ox.ac.uk/crystal/ukb/docs/primary_care_data.pdf for system suppliers, participant numbers and details of coding classifications used.

Selection process for included and excluded anti-anxiety/depressants

Selective serotonin reuptake inhibitors (SSRIs), for example citalopram, escitalopram, fluoxetine, paroxetine and sertraline, the recommended first line medications for anxiety and/or depression in primary care[1],[2] were included in our study alongside other commonly prescribed anti-anxiety/depressant medications i.e. the serotonin-norepinephrine reuptake inhibitors (SNRIs) for example, venlafaxine and duloxetine, the alpha-2 antagonists for example, mirtazapine and specific tricyclic anti-depressants (TCAs) for example dosulepin and lofepramine. The TCAs more commonly prescribed for neuropathic pain and fibromyalgia e.g. amitriptyline and monoamine oxidase inhibitors (MAOIs) were excluded as were mood stabilisers and anti-psychotic medications, as these are reserved for severe mental illnesses including bipolar disorder, schizophrenia and psychosis. Anxiolytics e.g. propranolol and diazepam were not included as these are more commonly prescribed on an 'as required' basis for temporary acute or phobic related anxiety presentations, rather than persisting anxiety symptoms for which regular, daily SSRIs, notably sertraline, would be the treatment choice.

[1]BNF: *Antidepressant drugs*:
[2]NICE: *First-choice antidepressant use in adults with depression or generalised anxiety disorder*
Key therapeutic topic. Published: 15 January 2015 nice.org.uk/guidance/ktt8

Development of anxiety and depression diagnosis and prescription code lists

Clinical events in the dataset are recorded as Read v2 or CTV3 codes[1]. Prescriptions were recorded as drug name, read v2, BNF and/or dm+d codes. Using the code lists provided by UK Biobank, together with recognised clinical/medication code repositories from other studies including CALIBER and clinicalcodes.org, we created anxiety and depression diagnosis and prescription code lists.

Supplementary Table S2a: Anxiety diagnosis code list

Read v2 code	CTV3 code	Description
2258.		O/E - anxious
225J.	XaKVA	O/E - panic attack
E200.		Anxiety states
E2000		Anxiety state unspecified
E2001	XE1Y7<br>Ua1qS	Panic disorder
E2002		Generalised anxiety disorder
E2003	X00Sb	Anxiety with depression
E2004		Chronic anxiety
E2005		Recurrent anxiety
E200z		Anxiety state NOS
E205.	XE1YD<br>XE0qk	Neurasthenia - nervous debility
		Nervous exhaustion
E28..	XE1Ym<br>Xa18j	Acute reaction to stress
E280.		Acute panic state due to acute stress reaction
E283.		Other acute stress reactions
E283z		Other acute stress reaction NOS
E284.		Stress reaction causing mixed disturbance of emotion
E28z.	XE1Yn	Acute stress reaction NOS
Eu054	X00RP	[X]Organic anxiety disorder
Eu341	E2112	[X]Dysthymia, [X]Persistant anxiety depression
Eu41.		[X]Other anxiety disorders

Eu410		[X]Panic disorder [episodic paroxysmal anxiety]
Eu411	E2002<br>E200.	[X]Generalized anxiety disorder
Eu412	X00Sb	[X]Mixed anxiety and depressive disorder
Eu413		[X]Other mixed anxiety disorders
Eu41y	XE1Zj	[X]Other specified anxiety disorders
Eu41z	E200.	[X]Anxiety disorder, unspecified
Eu430	XE1Ym	[X]Acute stress reaction

Supplementary Table S2b: Depression diagnosis code list

Read v2 code	CTV3 code	Read v2 Description
1B17.	XE0re<br>XM0CR<br>XM0CS<br>X00SO	Depressed
1BT..	XE0re	Depressed mood
2257.	2257.	O/E - depressed
62T1.	62T1.	Puerperal depression
9kQ..	XaPKm	On full dose long term treatment depression - enh serv admin
E112.	XE1Y0<br>X00SQ<br>X00SR<br>X00SS	Single major depressive episode
E1120		Single major depressive episode, unspecified
E1121		Single major depressive episode, mild
E1122		Single major depressive episode, moderate
E1123		Single major depressive episode, severe, without psychosis
E1124<br>E1125<br>E1126		Single major depressive episode, severe, with psychosis
E112z		Single major depressive episode NOS
E113.	XE1Y1	Recurrent major depressive episode

	XM1GC	
E1130		Recurrent major depressive episodes, unspecified
E1131		Recurrent major depressive episodes, mild
E1132		Recurrent major depressive episodes, moderate
E1133		Recurrent major depressive episodes, severe, no psychosis
E1134		Recurrent major depressive episodes, severe, with psychosis
E1135		
E1136		
E1137		
E113z		Recurrent major depressive episode NOS
E11y2		Atypical depressive disorder
E11z2		Masked depression
E135.		Agitated depression
E2003	X00Sb	Anxiety with depression
E204.	XE1YC	Neurotic depression reactive type
E291.		Prolonged depressive reaction
E2B..		Depressive disorder NEC
E2B1.		Chronic depression
Eu32.	XE1Y0	[X]Depressive episode
	XE1Zb	
	XE1YC	
Eu320		[X]Mild depressive episode
Eu321		[X]Moderate depressive episode
Eu322	XE1ZY	[X]Severe depressive episode without psychotic symptoms
	XaCHr	
	XaCHs	
Eu323	XE1ZZ	[X]Severe depressive episode with psychotic symptoms
	E1124	
Eu324	XaCIs	[X]Mild depression
Eu325	XSGok	[X]Major depression, mild
Eu326	XSGol	[X]Major depression, moderately severe
Eu327	XSGom	[X]Major depression, severe without psychotic symptoms
Eu328	XSGon	[X]Major depression, severe with psychotic symptoms
Eu329	XaX53	[X]Single major depr ep, severe with psych, psych in remiss
Eu32A	XaX54	[X]Recurr major depr ep, severe with psych, psych in remiss
Eu32B	XaY2C	[X]Antenatal depression
Eu32y	XE1Za	[X]Other depressive episodes
	X00SU	
Eu32z	XE1Zb	[X]Depressive episode, unspecified
	XE1YC	
	XaB9J	

Eu33.	XE1Zc<br>E1137<br>E130.<br>X761L	[X]Recurrent depressive disorder
Eu330		[X]Recurrent depressive disorder, current episode mild
Eu331		[X]Recurrent depressive disorder, current episode moderate
Eu332	XE1Zd<br>E1133<br>XaCHo	[X]Recurr depress disorder cur epi severe without psyc sympt
Eu333	XE1Ze<br>E1134	[X]Recurrent depress disorder cur epi severe with psyc symp
Eu334		[X]Recurrent depressive disorder, currently in remission
Eu33y		[X]Other recurrent depressive disorders
Eu33z	XE1Zf<br>X00SO	[X]Recurrent depressive disorder, unspecified
Eu412	X00Sb	[X]Mixed anxiety and depressive disorder
Eu530	62T1.	[X]Postnatal depression NOS
Eu34.	Eu34.	[X]Persistent mood affective disorders
Eu34y		[X]Other persistent mood affective disorders
Eu34z		[X]Persistent mood affective disorder, unspecified
Eu33z	XE1Zf	X]Recurrent depressive disorder, unspecified
Eu340	E2113	[X]Cyclothymia
Eu341	E2112	[X]Dysthymia
Eu3y.		[X]Other mood affective disorders
Eu3y0	XE1Zg	[X]Other single mood affective disorders
	Eu316	
Eu3y1	XE1Zh	[X]Other recurrent mood affective disorders
	Xa0wV	
Eu3yy		[X]Other specified mood affective disorders
Eu3z.		[X]Unspecified mood affective disorder
E290.<br>E290z		Brief depressive reaction

[1] NHS Digital: Read codes https://digital.nhs.uk/services/terminology-and-classifications/read-codes

Supplementary Table S2c: Anti-anxiety/depressant prescription code list

Code	
CITALOPRAM	
da9..	CITALOPRAM
da91.	CITALOPRAM 20mg tablets
da92.	CIPRAMIL 20mg tablets
da93.	CITALOPRAM 10mg tablets
da94.	*CIPRAMIL 10mg tablets
da95.	CITALOPRAM 40mg tablets
da96.	*CIPRAMIL 40mg tablets
da97.	CIPRAMIL 40mg/mL oral drops 15mL
da98.	*PAXORAN 10mg tablets
da99.	*PAXORAN 20mg tablets
da9A.	*PAXORAN 40mg tablets
da9z.	CITALOPRAM 40mg/mL oral drops
SERTRALINE	
da5..	SERTRALINE HYDROCHLORIDE
da51.	SERTRALINE 50mg tablets

da52. SERTRALINE 100mg tablets

da53. LUSTRAL 50mg tablets

da54. LUSTRAL 100mg tablets

FLUVOXAMINE

da3.. FLUVOXAMINE MALEATE

da31. FAVERIN 50mg tablets

da32. FLUVOXAMINE MALEATE 50mg tablets

da33. FAVERIN 100mg tablets

da34. FLUVOXAMINE MALEATE 100mg tablets

FLUOXETINE

da4.. FLUOXETINE HYDROCHLORIDE

da41. FLUOXETINE 20mg capsules

da42. *PROZAC 20mg capsules x30

da43. FLUOXETINE 20mg/5mL oral liquid

da44. PROZAC 20mg/5mL oral liquid

da45. PROZAC 20mg capsules

da46. FLUOXETINE 60mg capsules

da47. *PROZAC 60mg capsules

da48. *FELICIUM 20mg capsules

da49. OXACTIN 20mg capsules

da4A. RANFLUTIN 20mg capsules

da4B. PROZIT 20mg/5mL oral solution

da4C. PROZEP 20mg/5mL oral solution

da4D. OLENA 20mg dispersible tablets

da4E. FLUOXETINE 20mg dispersible tablets

PAROXETINE

da6.. PAROXETINE HYDROCHLORIDE

da61. PAROXETINE 20mg tablets

da62. SEROXAT 20mg tablets x30

da63. PAROXETINE 30mg tablets

da64. SEROXAT 30mg tablets x30

da65. PAROXETINE 10mg/5mL sugar free liquid

da66. SEROXAT 10mg/5mL sugar free liquid

da67. PAROXETINE 10mg tablets

da68. SEROXAT 10mg tablets

MIRTAZAPINE

daB..	MIRTAZAPINE
daB1.	MIRTAZAPINE 30mg tablets
daB2.	*ZISPIN 30mg tablets
daB3.	MIRTAZAPINE 30mg oro-dispersible tablets
daB4.	ZISPIN SOLTAB 30mg oro-dispersible tablets
daB5.	MIRTAZAPINE 15mg oro-dispersible tablets
daB6.	ZISPIN SOLTAB 15mg oro-dispersible tablets
daB7.	MIRTAZAPINE 45mg oro-dispersible tablets
daB8.	ZISPIN SOLTAB 45mg oro-dispersible tablets
daBy.	MIRTAZAPINE 45mg tablets
daBz.	MIRTAZAPINE 15mg tablets
DOSULEPIN/DOTHIEPIN/ PROTHIADEN	
d75..	DOSULEPIN HYDROCHLORIDE
d751.	PROTHIADEN 25mg capsules
d752.	PROTHIADEN 75mg tablets
d753.	*PREPADINE 25mg capsules
d754.	*PREPADINE 75mg tablets
d755.	*DOTHAPAX 25mg capsules

d756.	*DOTHAPAX 75mg tablets
d759.	*THADEN 25mg capsules
d75A.	*THADEN 75mg tablets
d75y.	DOSULEPIN HYDROCHLORIDE 25mg capsules
d75z.	DOSULEPIN HYDROCHLORIDE 75mg tablets
LOFEPRAMINE	
d79..	LOFEPRAMINE
d791.	*GAMANIL 70mg tablets x56CP
d792.	LOMONT 70mg/5mL sugar free suspension
d793.	*FEPRAPAX 70mg tablets
d794.	*GAMANIL 70mg tablets
d79y.	LOFEPRAMINE 70mg/5mL sugar free suspension
d79z.	LOFEPRAMINE 70mg tablets
VENLAFAXINE	
da7..	VENLAFAXINE
da71.	VENLAFAXINE 37.5mg tablets
da72.	VENLAFAXINE 75mg tablets
da73.	*EFEXOR 37.5mg tablets

da74.	*EFEXOR 75mg tablets
da75.	*VENLAFAXINE 50mg tablets
da76.	*EFEXOR 50mg tablets
da77.	VENLAFAXINE 75mg m/r capsules
da78.	EFEXOR XL 75mg m/r capsules
da79.	VENLAFAXINE 150mg m/r capsules
da7a.	VENAXX XL 150mg m/r capsules
da7A.	EFEXOR XL 150mg m/r capsules
da7b.	VAXALIN XL 75mg m/r capsules
da7B.	RODOMEL XL 75mg m/r capsules
da7c.	VAXALIN XL 150mg m/r capsules
da7C.	RODOMEL XL 150mg m/r capsules
da7d.	ALVENTA XL 75mg m/r capsules
da7D.	WINFEX XL 75mg m/r capsules
da7e.	ALVENTA XL 150mg m/r capsules
da7E.	WINFEX XL 150mg m/r capsules
da7f.	RANFAXINE XL 150mg m/r capsules
da7F.	TRIXAT XL 75mg m/r capsules

da7g.	RANFAXINE XL 75mg m/r capsules
da7G.	TRIXAT XL 150mg m/r capsules
da7h.	BONILUX XL 75mg m/r capsules
da7H.	VIEPAX XL 75mg m/r tablets
da7i.	BONILUX XL 150mg m/r capsules
da7I.	VENLAFAXINE 75mg m/r tablets
da7j.	TONPULAR XL 75mg m/r capsules
da7J.	VIEPAX XL 150mg m/r tablets
da7k.	TONPULAR XL 150mg m/r capsules
da7K.	VENLAFAXINE 150mg m/r tablets
da7l.	FORAVEN XL 75mg m/r capsules
da7L.	*TARDCAPS XL 75mg m/r capsules
da7m.	FORAVEN XL 150mg m/r capsules
da7M.	*TARDCAPS XL 150mg m/r capsule
da7n.	DEPEFEX XL 75mg m/r capsules
da7N.	VIEPAX 37.5mg tablets
da7o.	DEPEFEX XL 150mg m/r capsules
da7O.	VIEPAX 75mg tablets

da7p.	VENLALIC XL 37.5mg m/r tablets
da7P.	VENSIR XL 75mg m/r capsules
da7q.	VENLAFAXINE 37.5mg m/r tablets
da7Q.	VENSIR XL 150mg m/r capsules
da7r.	SUNVENIZ XL 75mg m/r tablets
da7R.	TIFAXIN XL 75mg m/r capsules
da7s.	SUNVENIZ XL 150mg m/r tablets
da7S.	TIFAXIN XL 150mg m/r capsules
da7t.	VENLADEX XL 75mg m/r tablets
da7T.	VEXARIN XL 75mg m/r capsules
da7u.	VENLADEX XL 150mg m/r tablets
da7U.	VEXARIN XL 150mg m/r capsules
da7v.	EFEXOR XL 225mg m/r capsules
da7V.	VENLALIC XL 75mg m/r tablets
da7w.	VENLAFAXINE 225mg m/r capsules
da7W.	VENLALIC XL 150mg m/r tablets
da7X.	VENLALIC XL 225mg m/r tablets
da7Y.	VENLAFAXINE 225mg m/r tablets

da7Z.	VENAXX XL 75mg m/r capsules
ESCITALOPRAM	
daC..	ESCITALOPRAM
daC1.	ESCITALOPRAM 10mg tablets
daC2.	CIPRALEX 10mg tablets
daC3.	ESCITALOPRAM 20mg tablets
daC4.	CIPRALEX 20mg tablets
daC5.	ESCITALOPRAM 5mg tablets
daC6.	CIPRALEX 5mg tablets
daC7.	ESCITALOPRAM 10mg/mL oral drops
daC8.	*CIPRALEX 10mg/mL oral drops
daC9.	CIPRALEX 20mg/mL oral drops
daCA.	ESCITALOPRAM 20mg/mL oral drops

Supplementary Table S3. Incident cases post Biobank baseline and prevalent cases pre-biobank baseline adjusted for pre-existing anxiety/depression diagnosis or prescription, complete case analysis.

Cox proportional hazard models of the association between socio-demographic, health, lifestyle and work characteristics and incident anxiety/depression in a) all IT workers compared to all other employed Biobank participants b) IT worker subgroups (managers, professionals, technicians) and c) IT subgroups compared to other similar occupations within their SOC tree (Functional Managers, Science and Technology Professionals, Science and Technology Associate Professionals).

Longitudinal study population: all employed Biobank participants with linked GP records

		**Model 0** [d]	**Model 1** [e]	**Model 2** [f]	**Model 3** [g]	**Model 4** [h]
		Unadjusted HR (95% CI)	Adjusted HR (95% CI)	Adjusted HR (95% CI)	Adjusted HR (95% CI)	Adjusted HR (95% CI)
**a**	Failures 21,035					
	All other employed participants Incidence rate* (2.4)	1.00	1.00	1.00	1.00	1.00
	All IT workers Incidence rate* (1.8)	0.76 (0.70-0.84)	0.85 (0.78-0.93)	0.92 (0.84-1.01)	0.93 (0.85-1.02)	0.96 (0.88-1.05)
**b**	Failures 504					
	IT managers Incidence rate* (1.8)	1.00	1.00	1.00	1.00	1.00
	IT professionals Incidence rate* (1.7)	0.98 (0.81-1.19)	1.01 (0.84-1.23)	0.95 (0.78-1.16)	0.96 (0.79-1.18)	0.97 (0.79-1.18)
	IT technicians Incidence rate* (2.2)	1.24 (0.95-1.61)	1.18 (0.91-1.54)	0.99 (0.75-1.31)	1.00 (0.76-1.32)	0.95 (0.72-1.27)
**c**	Failures 767					
	All other Functional managers Incidence rate* (1.9)	1.00	1.00	1.00	1.00	1.00
	IT managers Incidence rate* (1.8)	0.94 (0.79-1.11)	1.00 (0.84-1.18)	1.00 (0.84-1.18)	0.98 (0.83-1.16)	1.03 (0.87-1.23)
	Failures 576					
	All other Science & Technology professionals Incidence rate* (1.4)	1.00	1.00	1.00	1.00	1.00
	IT professionals Incidence rate* (1.7)	1.18 (1.00-1.39)	1.08 (0.91-1.28)	1.09 (0.92-1.29)	1.07 (0.91-1.27)	1.11 (0.92-1.35)
	Failures 250					

	All other Science & Technology Associate professionals<br>Incidence rate* (1.9)	1.00	1.00	1.00	1.00	1.00
	IT technicians<br>Incidence rate* (2.2)	1.15<br>(0.88-1.50)	1.04<br>0.80-1.36)	1.05<br>(0.80-1.38)	1.07<br>(0.81-1.40)	1.09<br>(0.82-1.46)

HR, hazard ratio; CI, confidence interval.
Model 0 [d] = Unadjusted
Model 1 [e] = Model 0 + sociodemographic factors i.e., confounders (age, sex, ethnicity, date of Biobank recruitment/baseline assessment, pre-existing anxiety/depression diagnosis or script)
Model 2 [f] = Model 1 + additional sociodemographic factors i.e., potential mediators (deprivation, annual household income, pre-existing anxiety/depression diagnosis or script)
Model 3 [g] = Model 2 + health and lifestyle factors (smoking, sleep duration, BMI, longstanding illness/disability, pre-existing anxiety/depression diagnosis or prescription).
Model 4 [h] = Model 3 + work-related factors (tenure in current occupation, working hours, work-related sedentary behaviour, shift work, pre-existing anxiety/depression diagnosis or prescription).
*Rates are expressed per 100 and based on person-years

Supplementary Table S4. Incident cases only post biobank baseline, complete case analysis.

Cox proportional hazard models of the association between socio-demographic, health, lifestyle and work characteristics and incident anxiety/depression in a) all IT workers compared to all other employed Biobank participants b) IT worker subgroups (managers, professionals, technicians) and c) IT subgroups compared to other similar occupations within their SOC tree (Functional Managers, Science and Technology Professionals, Science and Technology Associate Professionals).

Longitudinal study population: all employed Biobank participants with linked GP records

		Model 0 [d]	Model 1 [e]	Model 2 [f]	Model 3 [g]	Model 4 [h]
		Unadjusted HR (95% CI)	Adjusted HR (95% CI)	Adjusted HR (95% CI)	Adjusted HR (95% CI)	Adjusted HR (95% CI)
**a**	Failures 11,983					
	All other employed participants<br>Incidence rate* (2.2)	1.00	1.00	1.00	1.00	1.00
	All IT workers<br>Incidence rate* (1.7)	0.75<br>(0.68-0.83)	0.84<br>(0.76-0.93)	0.92<br>(0.83-1.02)	0.93<br>(0.83-1.03)	0.98<br>(0.88-1.09)
**b**	Failures 376					
	IT managers<br>Incidence rate* (1.7)	1.00	1.00	1.00	1.00	1.00
	IT professionals<br>Incidence rate* (1.5)	0.86<br>(0.69-1.08)	0.90<br>(0.72-1.12)	0.84<br>(0.67-1.06)	0.85<br>(0.67-1.06)	0.86<br>(0.68-1.08)
	IT technicians<br>Incidence rate* (2.0)	1.17<br>(0.86-1.59)	1.12<br>(0.82-1.53)	0.95<br>(0.68-1.32)	0.95<br>(0.68-1.32)	0.89<br>(0.63-1.24)

**c**	Failures 578					
	All other Functional managers<br>Incidence rate* (1.8)<br><br>IT managers<br>Incidence rate* (1.7)	1.00<br><br><br>0.98<br>(0.81-1.19)	1.00<br><br><br>1.05<br>(0.86-1.27)	1.00<br><br><br>1.05<br>(0.86-1.28)	1.00<br><br><br>1.03<br>(0.85-1.26)	1.00<br><br><br>1.08<br>(0.89-1.31)
	Failures 404					
	All other Science & Technology professionals<br>Incidence rate* (1.3)<br><br>IT professionals<br>Incidence rate* (1.5)	1.00<br><br><br><br>1.12<br>(0.92-1.36)	1.00<br><br><br><br>1.03<br>(0.84-1.26)	1.00<br><br><br><br>1.02<br>(0.84-1.25)	1.00<br><br><br><br>1.01<br>(0.83-1.23)	1.00<br><br><br><br>1.07<br>(0.86-1.35)
	Failures 168					
	All other Science & Technology Associate professionals<br>Incidence rate* (1.7)<br><br>IT technicians<br>Incidence rate* (2.0)	1.00<br><br><br><br>1.19<br>(0.87-1.64)	1.00<br><br><br><br>1.10<br>(0.80-1.52)	1.00<br><br><br><br>1.07<br>(0.78-1.49)	1.00<br><br><br><br>1.09<br>(0.79-1.51)	1.00<br><br><br><br>1.17<br>(0.82-1.67)

HR, hazard ratio; CI, confidence interval.
Model 0 [d] = Unadjusted
Model 1 [e] = Model 0 + sociodemographic factors i.e., confounders (age, sex, ethnicity, date of Biobank recruitment/baseline assessment)
Model 2 [f] = Model 1 + additional sociodemographic factors i.e., potential mediators (deprivation, annual household income)
Model 3 [g] = Model 2 + health and lifestyle factors (smoking, sleep duration, BMI, longstanding illness/disability).
Model 4 [h] = Model 3 + work-related factors (tenure in current occupation, working hours, work-related sedentary behaviour, shift work).
*Rates are expressed per 100 and based on person-years

Supplementary Table S5. Incident cases stratified by age "40-55" and "55+" year groups, complete case analysis.

Cox proportional hazard models of the association between socio-demographic, health, lifestyle and work characteristics and incident anxiety/depression in a) all IT workers compared to all other employed Biobank participants b) IT worker subgroups (managers, professionals, technicians) and c) IT subgroups compared to other similar occupations within their SOC tree (Functional Managers, Science and Technology Professionals, Science and Technology Associate Professionals).

Longitudinal study population: all employed Biobank participants with linked GP records

		Model 0 [d]		Model 1 [e]		Model 2 [f]		Model 3 [g]		Model 4 [h]	
		Unadjusted HR (95% CI)		Adjusted HR (95% CI)		Adjusted HR (95% CI)		Adjusted HR (95% CI)		Adjusted HR (95% CI)	
**a**	**Age (years)**	**40-55**	**55+**	**40-55**	**55+**	**40-55**	**55+**	**40-55**	**55+**	**40-55**	**55+**
	Failures 40-55 years<br>Failures 55+ years	8,414<br>3569									
	All other employed participants	1.00	1.00	1.00	1.00	1.00	1.00	1.00	1.00	1.00	1.00
	Incidence rate 40-55 years* (2.5)<br>Incidence rate 55+ years* (1.7)										
	All IT workers	0.72 (0.64-0.81)	0.67 (0.52-0.86)	0.86 (0.77-0.97)	0.76 (0.59- 0.98)	0.95 (0.85-1.07)	0.83 (0.64-1.06)	0.96 (0.85-1.07)	0.83 (0.64-1.06)	1.01 (0.90- 1.14)	0.86 (0.67-1.11)
	Incidence rate 40-55 years* (1.8)<br>Incidence rate 55+ years* (1.2)										
**b**	Failures 40-55 years<br>Failures 55+ years	313<br>63									
	IT managers	1.00	1.00	1.00	1.00	1.00	1.00	1.00	1.00	1.00	1.00
	Incidence rate 40-55 years* (1.9)<br>Incidence rate 55+ years* (1.2)										
	IT professionals	0.86 (0.62- 1.09)	0.93 (0.54- 1.60)	0.88 (0.69- 1.13)	0.95 (0.55-1.65)	0.84 (0.65- 1.08)	0.87 (0.49-1.54)	0.85 (0.66-1.09)	0.88 (0.50-1.56)	0.87 (0.68-1.13)	0.87 (0.48-1.57)
	Incidence rate 40-55 years* (1.7)<br>Incidence rate 55+ years* (1.2)										
	IT technicians	1.28 (0.91-1.78)	0.91 (0.42-2.00)	1.18 (0.84-1.66)	0.86 (0.39-1.88)	1.00 (0.70- 1.44)	0.72 (0.32-1.63)	1.00 (0.70-1.44)	0.71 (0.31-1.62)	0.95 (0.68 -1.38)	0.54 (0.23-1.30)
	Incidence rate 40-55 years* (2.4)<br>Incidence rate 55+ years* (1.1)										
**c**	Failures 40-55 years	464<br>114									

	Failures 55+ years										
	All other Functional managers	1.00	1.00	1.00	1.00	1.00	1.00	1.00	1.00	1.00	1.00
	Incidence rate 40-55 years* (2.0)<br>Incidence rate 55+ years* (1.3)										
	IT managers	0.95<br>(0.77-1.18)	0.95<br>(0.59-1.52)	1.07<br>(0.86-1.33)	0.95<br>(0.59- 1.53)	1.07<br>(0.86-1.33)	0.97<br>(0.60- 1.56)	1.05<br>(0.85-1.31)	0.94<br>(0.58-1.52)	1.09<br>(0.88-1.36)	0.93<br>(0.57-1.51)
	Incidence rate 40-55 years* (1.9)<br>Incidence rate 55+ years* (1.2)										
	Failures 40-55 years<br>Failures 55+ years	311<br>93									
	All other Science & Technology professionals	1.00	1.00	1.00	1.00	1.00	1.00	1.00	1.00	1.00	1.00
	Incidence rate 40-55 years* (1.5)<br>Incidence rate 55+ years* (0.99)										
	IT professionals	1.04<br>(0.83-1.29)	1.14<br>(0.75-1.74)	1.02<br>(0.82 -1.28)	1.06<br>(0.69-1.64)	1.03<br>(0.82 -1.29)	1.01<br>(0.65-1.57)	1.01<br>(0.81-1.27)	0.94<br>(0.60-1.48)	1.08<br>(0.83-1.4)	1.00<br>(0.61-1.64)
	Incidence rate 40-55 years* (1.7)<br>Incidence rate 55+ years* (1.2)										
	Failures 40-55 years<br>Failures 55+ years	121<br>47									
	All other Science & Technology Associate professionals	1.00	1.00	1.00	1.00	1.00	1.00	1.00	1.00	1.00	1.00
	Incidence rate 40-55 years* (1.9)<br>Incidence rate 55+ years* (1.4)										
	IT technicians	1.30	0.74	1.25	0.71	1.26	0.68	1.26	0.84	1.31	1.04

	Incidence rate 40-55 years* (2.4)<br>Incidence rate 55+ years* (1.1)	(0.90-1.87)	(0.36-1.54)	(0.86-1.80)	(0.34-1.48)	(0.87-1.82)	(0.33-1.44)	(0.87 -1.83)	(0.39-1.79)	(0.87-1.95)	(0.47-2.30)

HR, hazard ratio; CI, confidence interval.
Model 0 [d] = Unadjusted
Model 1 [e] = Model 0 + sociodemographic factors i.e., confounders (age, sex, ethnicity, date of Biobank recruitment/baseline assessment)
Model 2 [f] = Model 1 + additional sociodemographic factors i.e., potential mediators (deprivation, annual household income)
Model 3 [g] = Model 2 + health and lifestyle factors (smoking, sleep duration, BMI, longstanding illness/disability).
Model 4 [h] = Model 3 + work-related factors (tenure in current occupation, working hours, work-related sedentary behaviour, shift work).
*Rates are expressed per 100 and based on person-years

Supplementary Table S6. Incident cases for anxiety/depression diagnosis only, complete case analysis.

Cox proportional hazard models of the association between socio-demographic, health, lifestyle and work characteristics and incident anxiety/depression in a) all IT workers compared to all other employed Biobank participants b) IT worker subgroups (managers, professionals, technicians) and c) IT subgroups compared to other similar occupations within their SOC tree (Functional Managers, Science and Technology Professionals, Science and Technology Associate Professionals).

Longitudinal study population: all employed Biobank participants with linked GP records

		**Model 0** [d]	**Model 1** [e]	**Model 2** [f]	**Model 3** [g]	**Model 4** [h]
	**ANXIETY/DEPRESSION DIAGNOSIS ONLY**					
		Unadjusted HR (95% CI)	Adjusted HR (95% CI)	Adjusted HR (95% CI)	Adjusted HR (95% CI)	Adjusted HR (95% CI)
**a**	Failures 12,136					
	All other employed participants Incidence rate* (1.6)	1.00	1.00	1.00	1.00	1.00
	All IT workers Incidence rate* (1.3)	0.79 (0.70-0.89)	0.87 (0.77-0.97)	0.96 (0.85-1.08)	0.96 (0.86-1.09)	1.01 (0.90-1.14)
**b**	Failures 294					
	IT managers Incidence rate* (1.3)	1.00	1.00	1.00	1.00	1.00
	IT professionals Incidence rate* (1.2)	0.92 (0.71-1.18)	0.96 (0.74-1.23)	0.90 (0,69-1.16)	0.90 (0.69-1.17)	0.91 (0.70-1.19)
	IT technicians	1.14	1.09	0.93	0.93	0.86

	Incidence rate* (1.4)	(0.80-1.63)	(0.76-1.56)	(0.64-1.36)	(0.64-1.36)	(0.58-1.26)
**c**	Failures 431					
	All other Functional managers<br>Incidence rate* (1.3)<br><br>IT managers<br>Incidence rate* (1.3)	1.00<br><br><br>1.01<br>(0.81-1.26)	1.00<br><br><br>1.07<br>(0.85-1.34)	1.00<br><br><br>1.06<br>(0.85-1.33)	1.00<br><br><br>1.05<br>(0.83-1.31)	1.00<br><br><br>1.08<br>(0.86-1.36)
	Failures 318					
	All other Science & Technology professionals<br>Incidence rate* (0.9)<br><br>IT professionals<br>Incidence rate* (1.2)	1.00<br><br><br><br>1.18<br>(0.94-1.47)	1.00<br><br><br><br>1.08<br>(0.86-1.35)	1.00<br><br><br><br>1.07<br>(0.86-1.34)	1.00<br><br><br><br>1.06<br>(0.85-1.33)	1.00<br><br><br><br>1.14<br>(0.88-1.47)
	Failures 112					
	All other Science & Technology Associate professionals<br>Incidence rate* (1.1)<br><br>IT technicians<br>Incidence rate* (1.4)	1.00<br><br><br><br>1.41<br>(0.97-2.07)	1.00<br><br><br><br>1.28<br>(0.87-1.88)	1.00<br><br><br><br>1.26<br>(0.85-1.86)	1.00<br><br><br><br>1.29<br>(0.87-1.92)	1.00<br><br><br><br>1.26<br>(0.82-1.93)

HR, hazard ratio; CI, confidence interval.
Model 0 [d] = Unadjusted
Model 1 [e] = Model 0 + sociodemographic factors i.e., confounders (age, sex, ethnicity, date of Biobank recruitment/baseline assessment)
Model 2 [f] = Model 1 + additional sociodemographic factors i.e., potential mediators (deprivation, annual household income)
Model 3 [g] = Model 2 + health and lifestyle factors (smoking, sleep duration, BMI, longstanding illness/disability).
Model 4 [h] = Model 3 + work-related factors (tenure in current occupation, working hours, work-related sedentary behaviour, shift work).
*Rates are expressed per 100 and based on person-years

Supplementary Table S7. Incident cases for anxiety/depression prescriptions only, complete case analysis.

Cox proportional hazard models of the association between socio-demographic, health, lifestyle and work characteristics and incident anxiety/depression in a) all IT workers compared to all other employed Biobank participants b) IT worker subgroups (managers, professionals, technicians) and c) IT subgroups compared to other similar occupations within their SOC tree (Functional Managers, Science and Technology Professionals, Science and Technology Associate Professionals).

Longitudinal study population: all employed Biobank participants with linked GP records

		Model 0 [d]	Model 1 [e]	Model 2 [f]	Model 3 [g]	Model 4 [h]
	**ANXIETY/DEPRESSION PRESCRIPTIONS ONLY**					
		Unadjusted HR (95% CI)	Adjusted HR (95% CI)	Adjusted HR (95% CI)	Adjusted HR (95% CI)	Adjusted HR (95% CI)
**a**	Failures 9,680					
	All other employed participants Incidence rate* (1.7)	1.00	1.00	1.00	1.00	1.00
	All IT workers Incidence rate* (1.3)	0.70 (0.62-0.78)	0.78 (0.70-0.88)	0.87 (0.77-0.98)	0.87 (0.77-0.98)	0.92 (0.81-1.04)
**b**	Failures 282					
	IT managers Incidence rate* (1.4)	1.00	1.00	1.00	1.00	1.00
	IT professionals Incidence rate* (1.1)	0.73 (0.57-0.94)	0.76 (0.59-0.99)	0.71 (0.55-0.92)	0.71 (0.55-0.93)	0.72 (0.55-0.94)
	IT technicians Incidence rate* (1.6)	1.14 (0.80-1.60)	1.09 (0.77-1.54)	0.91 (0.62-1.31)	0.91 (0.63-1.32)	0.85 (0.58-1.24)
**c**	Failures 439					
	All other Functional managers Incidence rate* (1.3)	1.00	1.00	1.00	1.00	1.00
	IT managers Incidence rate* (1.4)	1.07 (0.86-1.33)	1.16 (0.93-1.44)	1.16 (0.93-1.44)	1.14 (0.91-1.42)	1.17 (0.93-1.46)
	Failures 292					
	All other Science & Technology professionals Incidence rate* (1.0)	1.00	1.00	1.00	1.00	1.00
	IT professionals Incidence rate* (1.1)	1.03 (0.82-1.30)	0.95 (0.75-1.20)	0.95 (0.75-1.20)	0.94 (0.74-1.18)	1.02 (0.78-1.34)
	Failures 136					

	All other Science & Technology Associate professionals<br>Incidence rate* (1.4)<br><br>IT technicians<br>Incidence rate* (1.6)	1.00<br><br><br><br>1.15<br>(0.81-1.64)	1.00<br><br><br><br>1.08<br>(0.75-1.54)	1.00<br><br><br><br>1.06<br>(0.73-1.52)	1.00<br><br><br><br>1.08<br>(0.75-1.56)	1.00<br><br><br><br>1.21<br>(0.82-1.79)

HR, hazard ratio; CI, confidence interval.
Model 0 [d] = Unadjusted
Model 1 [e] = Model 0 + sociodemographic factors i.e., confounders (age, sex, ethnicity, date of Biobank recruitment/baseline assessment)
Model 2 [f] = Model 1 + additional sociodemographic factors i.e., potential mediators (deprivation, annual household income)
Model 3 [g] = Model 2 + health and lifestyle factors (smoking, sleep duration, BMI, longstanding illness/disability).
Model 4 [h] = Model 3 + work-related factors (tenure in current occupation, working hours, work-related sedentary behaviour, shift work).
*Rates are expressed per 100 and based on person-years

Supplementary Table S8. Incident cases stratified by tenure in employment at baseline < 10 years and ≥ 10 years, complete case analysis. Cox proportional hazard models of the association between socio-demographic, health, lifestyle and work characteristics and incident anxiety/depression in a) all IT workers compared to all other employed Biobank participants b) IT worker subgroups (managers, professionals, technicians) and c) IT subgroups compared to other similar occupations within their SOC tree (Functional Managers, Science and Technology Professionals, Science and Technology Associate Professionals).

Longitudinal study population: all employed Biobank participants with linked GP records

		**Model 0 [d]**		**Model 1 [e]**		**Model 2 [f]**		**Model 3 [g]**		**Model 4 [h]**	
		Unadjusted HR (95% CI)		Adjusted HR (95% CI)		Adjusted HR (95% CI)		Adjusted HR (95% CI)		Adjusted HR (95% CI)	
**a**	**Tenure in employment (years)**	**< 10**	**≥ 10**	**< 10**	**≥ 10**	**< 10**	**≥ 10**	**< 10**	**≥ 10**	**< 10**	**≥ 10**
	Failures < 10 years<br>Failures ≥ 10 years	7,113<br>4,870									
	All other employed participants<br><br>Incidence rate < 10 years* (2.4)<br>Incidence rate ≥ 10 years* (1.9)	1.00	1.00	1.00	1.00	1.00	1.00	1.00	1.00	1.00	1.00
	All IT workers<br><br>Incidence rate	0.74<br>(0.65- 0.85)	0.78<br>(0.66- 0.90)	0.85<br>(0.74-0.98)	0.84<br>(0.72-0.98)	0.95<br>(0.83-1.10)	0.89<br>(0.77-1.04)	0.95<br>(0.83-1.10)	0.90<br>(0.77-1.06)	0.99<br>(0.86-1.14)	0.97<br>(0.83-1.13)

	< 10 years*<br>(1.8)<br>Incidence rate<br>≥ 10 years*<br>(1.6)										
**b**	Failures <<br>10years<br>Failures ≥ 10<br>years	207<br><br>169									
	IT managers<br><br>Incidence rate<br>< 10 years*<br>(1.8)<br>Incidence rate<br>≥ 10 years*<br>(1.7)	1.00	1.00	1.00	1.00	1.00	1.00	1.00	1.00	1.00	1.00
	IT professionals<br><br>Incidence rate<br>< 10 years*<br>(1.6)<br>Incidence rate<br>≥ 10 years*<br>(1.5)	0.90<br>(0.66-1.22)	0.83<br>(0.60-1.15)	0.94<br>(0.69-1.27)	0.84<br>(0.61-1.17)	0.87<br>(0.64-1.19)	0.79<br>(0.57-1.11)	0.87<br>(0.63- 1.19)	0.80<br>(0.57-1.13)	0.87<br>(0.63-1.20)	0.82<br>(0.58-1.16)
	IT technicians<br><br>Incidence rate<br>< 10 years*<br>(2.9)<br>Incidence rate<br>≥ 10 years*<br>(1.4)	1.67<br>(1.12-2.50)	0.79<br>(0.48-1.28)	1.50<br>(1.00-2.26)	0.79<br>(0.49-1.29)	1.26<br>(0.81-1.95)	0.68<br>(0.40-.1.13)	1.23<br>(0.79-1.90)	0.69<br>(0.41-1.15)	1.13<br>(0.72-1.77)	0.65<br>(0.38-1.10)
**c**	Failures <<br>10years<br>Failures ≥ 10<br>years	346<br><br>232									
	All other<br>Functional<br>managers<br><br>Incidence rate<br>< 10 years*<br>(1.9)<br>Incidence rate<br>≥ 10 years*<br>(1.7)	1.00	1.00	1.00	1.00	1.00	1.00	1.00	1.00	1.00	1.00
	IT managers<br><br>Incidence rate<br>< 10 years*<br>(1.8)<br>Incidence rate<br>≥ 10 years*<br>(1.7)	0.95<br>(0.73-1.24)	1.02<br>(0.77-1.36)	1.06<br>(0.81-1.38)	1.04<br>(0.78-1.39)	1.07<br>(0.82-1.40)	1.03<br>(0.77-1.38)	1.04<br>(0.80-1.37)	1.02<br>(0.76-1.37)	1.07<br>(0.81-1.40)	1.05<br>(0.78-1.42)

	Failures < 10years<br>Failures ≥ 10 years	225<br>179									
	All other Science & Technology professionals<br><br>Incidence rate < 10 years* (1.5)<br>Incidence rate ≥ 10 years* (1.1)	1.00	1.00	1.00	1.00	1.00	1.00	1.00	1.00	1.00	1.00
	IT professionals<br><br>Incidence rate < 10 years* (1.6)<br>Incidence rate ≥ 10 years* (1.5)	1.03 (0.79-1.34)	1.23 (0.91 -1.65)	0.93 (0.71-1.22)	1.15 (0.86-1.55)	0.93 (0.71-1.22)	1.12 (0.83-1.50)	0.90 (0.69-1.18)	1.12 (0.83-1.51)	0.94 (0.70-1.27)	1.23 (0.87-1.74)
	Failures < 10years<br>Failures ≥ 10 years	97<br>71									
	All other Science & Technology Associate professionals<br><br>Incidence rate < 10 years* (1.8)<br>Incidence rate ≥ 10 years* (1.6)	1.00	1.00	1.00	1.00	1.00	1.00	1.00	1.00	1.00	1.00
	IT technicians<br><br>Incidence rate < 10 years* (2.9)<br>Incidence rate ≥ 10 years* (1.4)	1.68 (1.11-2.54)	0.81 (0.49-1.34)	1.51 (1.00-2.29)	0.76 (0.46-1.27)	1.37 (0.89-2.10)	0.77 (0.46-1.29)	1.41 (0.92-2.16)	0.76 (0.45-1.28)	1.49 (0.94-2.38)	0.78 (0.45-1.37)

HR, hazard ratio; CI, confidence interval.
Model 0 [d] = Unadjusted
Model 1 [e] = Model 0 + sociodemographic factors i.e., confounders (age, sex, ethnicity, date of Biobank recruitment/baseline assessment)
Model 2 [f] = Model 1 + additional sociodemographic factors i.e., potential mediators (deprivation, annual household income)
Model 3 [g] = Model 2 + health and lifestyle factors (smoking, sleep duration, BMI, longstanding illness/disability).
Model 4 [h] = Model 3 + work-related factors (tenure in current occupation, working hours, work-related sedentary behaviour, shift work).
*Rates are expressed per 100 and based on person-years
