# Supplementary material for: Comparing Anxiety and Depression in Information Technology Workers with Others in Employment: A UK Biobank Cohort Study
Source: Ann Work Expo Health. 2022 Aug 27;66(9):1136–50. doi: 10.1093/annweh/wxac061 (PMC9664232; doi:10.1093/annweh/wxac061)
Supplement: Supplementary file 1 [file wxac061_suppl_supplementary_material.docx]

Title: Comparing anxiety and depression in Information Technology workers with others in employment: A UK Biobank cohort study

Author(s): Drushca Lalloo, Jim Lewsey, Srinivasa Vittal Katikireddi, Ewan B Macdonald, Desmond Campbell, Evangelia Demou.

**SUPPLEMENTARY MATERIAL** 

**Supplementary Table S1. Self-reported mental health and psychosocial characteristics in a) all IT workers compared to all other employed Biobank participants b) IT worker subgroups (managers, professionals, technicians) and c) IT subgroups compared to other similar occupations within their SOC tree (Functional Managers, Science and Technology Professionals, Science and Technology Associate Professionals)**

*Cross-sectional study population: all employed biobank participants at baseline* 

|  | a |  | b |  |  | c |  |  |
| --- | --- | --- | --- | --- | --- | --- | --- | --- |
|  | **All other employed Biobank participants** | **All IT workers** | **IT managers** | **IT professionals** | **IT technicians** | **All other Functional managers** | **All other Science &Technology professionals** | **All other Science & Technology Associate professionals** |
| Total n (%) 287,137 (100) | 276,207 (96.2) | 10,930 (3.8) | 3,698 (1.3) | 5,755 (2.0) | 1,477 (0.5) | 11,767 (4.1) | 8,158 (2.8) | 3,201 (1.1) |
| **Self-reported mental ill-health** Ever seen a GP for nerves, anxiety, tension or depression *No* *Yes* *Missing* Ever seen a psychiatrist for nerves, anxiety, tension or depression *No* *Yes* *Missing* **Self-reported psychological distress:** PHQ (4 items) Frequency of depressed mood in last 2 weeks *Not at all* *Several days* *More than half the days* *Nearly every day* *Missing* Frequency of unenthusiasm/ disinterest in last 2 weeks *Not at all* *Several days* *More than half the days* *Nearly every day* *Missing* Frequency of tenseness/ restlessness in last 2 weeks *Not at all* *Several days* *More than half the days* *Nearly every day* *Missing* Frequency of tiredness/lethargy in last 2 weeks *Not at all* *Several days* *More than half the days* *Nearly every day* *Missing* **Anxiety/depression symptoms** **PHQ total score (0-12)** 0-5 (No symptoms) 6-12 (Symptoms) *Missing* **UCLA Loneliness Scale** **(2 questions below)** Do you often feel lonely? *No* *Yes* *Missing* How often are you able to confide in someone close to you? *Almost daily to once every few months* *Never or almost never* *Missing* **UCLA Loneliness Scale** **Total score (0-2)** 0-1 (Not lonely) 2 (Lonely) *Missing* **Risk taking** Would you describe yourself as someone who takes risks? *No* *Yes* *Missing* | 185,453 (67.1) 88,897 (32.2) 1,857 (0.7) 248,115 (89.8) 26,966 (9.8) 1,126 (0.4) 200,838 (72.7) 51,681 (18.7) 7,694 (2.8) 4,131 (1.5) 11,863 (4.3) 208,705 (75.6) 45,532 (16.5) 7,421 (2.7) 4,839 (1.8) 9,710 (3.5) 193,457 (70) 61,192 (22.2) 7,183 (2.6) 4,162 (1.5) 10,213 (3.7) 118,402 (42.9) 116,578 (42.2) 17,941 (6.5) 15,735 (5.7) 7,551 (2.7) 247,493 (89.6) 2,421 (0.9) 26,293 (9.5) 223,180 (80.8) 48,536 (17.6) 4,491 (1.6) 231,990 (84) 36,200 (13.1) 8,017 (2.9) 228,650 (82.8) 35,670 (12.9) 11,887 (4.3) 185,911 (67.3) 79,963 (29) 10,333 (3.7) | 8,158 (74.6) 2,732 (25) 40 (0.4) 9,967 (91.2) 930 (8.5) 33 (0.3) 8,288 (75.8) 2,054 (18.8) 238 (2.2) 124 (1.1) 226 (2.1) 8,318 (76.1) 2,026 (18.5) 218 (2) 163 (1.5) 204 (1.9) 7,801 (71.4) 2,573 (23.5) 199 (1.8) 137 (1.3) 220 (2) 5,139 (47) 4,533 (41.5) 638 (5.8) 479 (4.4) 141 (1.3) 10,304 (94.3) 68 (0.6) 558 (5.1) 9,065 (82.9) 1,741 (15.9) 124 (1.1) 8,954 (81.9) 1,730 (15.8) 246 (2.3) 9,372 (85.8) 1,199 (11) 359 (3.3) 7,282 (66.6) 3,335 (30.5) 313 (2.9) | 2,772 (75) 913 (24.7) 13 (0.4) 3,403 (92) 288 (7.8) 7 (0.2) 2,861 (77.4) 674 (18.2) 64 (1.7) 43 (1.2) 56 (1.5) 2,863 (77.4) 658 (17.8) 65 (1.8) 43 (1.2) 69 (1.9) 2,613 (70.7) 898 (24.3) 72 (2) 52 (1.4) 63 (1.7) 1,740 (47.1) 1,551 (41.9) 217 (5.9) 158 (4.3) 32 (0.9) 3,523 (95.3) 20 (0.5) 155 (4.2) 3,155 (85.3) 508 (13.7) 35 (1) 3,088 (83.5) 563 (15.2) 47 (1.3) 3,252 (87.9) 366 (9.9) 80 (2.2) 2,266 (61.3) 1,354 (36.6) 78 (2.1) | 4,380 (76.1) 1,353 (23.5) 22 (0.4) 5,248 (91.2) 489 (8.5) 18 (0.3) 4,355 (75.7) 1,092 (19) 136 (2.4) 58 (1) 114 (2) 4,358 (75.7) 1,097 (19.1) 114 (2) 88 (1.5) 98 (1.7) 4,172 (72.5) 1,322 (23) 94 (1.6) 61 (1.1) 106 (1.8) 2,762 (48) 2,372 (41.2) 317 (5.5) 230 (4) 74 (1.3) 5,446 (94.6) 29 (0.5) 280 (4.5) 4,738 (82.3) 948 (16.5) 69 (1.2) 4,654 (80.9) 946 (16.4) 155 (2.7) 4,913 (85.4) 623 (10.8) 219 (3.8) 3,884 (67.5) 1,685 (29.3) 186 (3.2) | 1,006 (68.1) 466 (31.6) 5 (0.3) 1,316 (89.1) 153 (10.4) 8 (0.5) 1,072 (72.6) 288 (19.5) 38 (2.6) 23 (1.6) 56 (3.8) 1,098 (74.3) 271 (18.4) 39 (2.6) 32 (2.2) 37 (2.5) 1,016 (68.8) 353 (23.9) 33 (2.2) 24 (1.6) 51 (3.5) 637 (43.1) 610 (41.3) 104 (7) 91 (6.2) 35 (2.4) 1,335 (90.4) 19 (1.3) 123 (8.3) 1,172 (79.4) 285 (19.3) 20 (1.4) 1,212 (82.1) 221 (15) 44 (3) 1,207 (81.7) 210 (14.2) 60 (4.1) 1,132 (76.6) 296 (20) 49 (3.3) | 8,520 (72.4) 3,203 ((27.2) 44 (0.4) 10,751 (91.4) 998 (8.5) 18 (0.1) 9,134 (77.6) 2,037 (17.3) 223 (1.9) 113 (1.0) 260 (2.2) 9,400 (79.9) 1,802 (15.3) 203 (1.7) 151 (1.3) 211 (1.8) 8,339 (70.9) 2,854 (24.3) 210 (1.8) 150 (1.3) 214 (1.8) 5,511 (46.8) 5,004 (42.5) 626 (5.3) 451 (3.8) 175 (1.5) 11,081 (94.2) 70 (0.6) 616 (5.2) 10,038 (85.3) 1,632 (13.9) 97 (0.8) 10,154 (86.3) 1,423 (12.1) 190 (1.6) 10,267 (87.3) 1,222 (10.4) 278 (2.3) 6,840 (58.1) 4,635 (39.4) 292 (2.5) | 6,315 (77.4) 1,812 (22.2) 31 (0.4) 7,518 (92.2) 616 (7.6) 24 (0.2) 6,417 (78.7) 1,313 (16.1) 143 (1.8) 63 (0.8) 222 (2.7) 6,435 (78.9) 1,301 (16) 129 (1.6) 99 (1.2) 194 (2.4) 6,069 (74.4) 1,682 (20.6) 148 (1.8) 82 (1.0) 177 (2.2) 4,082 (50.0) 3,249 (39.8) 379 (4.7) 295 (3.6) 153 (1.9) 7,580 (92.9) 44 (0.5) 534 (6.6) 7,027 (86.1) 1,025 (12.6) 106 (1.3) 6619 (81.1) 1,327 (16.3) 212 (2.6) 7,153 (87.7) 701 (8.6) 304 (3.7) 5,348 (65.6) 2,553 (31.3) 257 (3.2) | 2,342 (73.2) 841 (26.3) 18 (0.5) 2,936 (91.7) 254 (7.9) 11 (0.3) 2,382 (74.4) 578 (18.1) 65 (2.0) 43 (1.3) 133 (4.2) 2,415 (75.5) 565 (17.7) 86 (2.7) 43 (1.3) 92 (2.9) 2,251 (70.3) 730 (22.8) 68 (2.1) 47 (1.5) 105 (3.3) 1,432 (44.7) 1,340 (41.9) 188 (5.9) 157 (4.9) 84 (2.6) 2,899 (90.6) 24 (0.8) 278 (8.6) 2,637 (82.4) 516 (16.1) 48 (1.5) 2543 (79.4) 549 (17.2) 109 (3.4) 2,699 (84.3) 347 (10.8) 155 (4.8) 2,335 (73.0) 744 (23.2) 122 (3.8) |

**UK Biobank linkage with GP dataset**

In 2019, coded primary care/GP data for approximately 45% of the UK Biobank cohort were linked to their records. This linkage was based on having a compatible GP system namely: EMIS and Vision (for Scotland and Wales) and TPP and Vision (for England). The GP dataset includes prescription and clinical event records, with corresponding dates. See [https://biobank.ctsu.ox.ac.uk/crystal/ukb/docs/primary_care_data.pdf](https://biobank.ctsu.ox.ac.uk/crystal/ukb/docs/primary_care_data.pdf) for system suppliers, participant numbers and details of coding classifications used.

**Selection process for included and excluded anti-anxiety/depressants**

Selective serotonin reuptake inhibitors (SSRIs), for example citalopram, escitalopram, fluoxetine, paroxetine and sertraline, the recommended first line medications for anxiety and/or depression in primary care^1^,^2^ were included in our study alongside other commonly prescribed anti-anxiety/depressant medications i.e. the serotonin-norepinephrine reuptake inhibitors (SNRIs) for example, venlafaxine and duloxetine, the alpha-2 antagonists for example, mirtazapine and specific tricyclic anti-depressants (TCAs) for example dosulepin and lofepramine. The TCAs more commonly prescribed for neuropathic pain and fibromyalgia e.g. amitriptyline and monoamine oxidase inhibitors (MAOIs) were excluded as were mood stabilisers and anti-psychotic medications, as these are reserved for severe mental illnesses including bipolar disorder, schizophrenia and psychosis. Anxiolytics e.g. propranolol and diazepam were not included as these are more commonly prescribed on an ‘as required’ basis for temporary acute or phobic related anxiety presentations, rather than persisting anxiety symptoms for which regular, daily SSRIs, notably sertraline, would be the treatment choice.

^1^BNF: *Antidepressant drugs*: 

^2^NICE: *First-choice antidepressant use in adults with depression or generalised anxiety disorder*

*Key therapeutic topic.* Published: 15 January 2015 nice.org.uk/guidance/ktt8

**Development of anxiety and depression diagnosis and prescription code lists**

Clinical events in the dataset are recorded as Read v2 or CTV3 codes^1^. Prescriptions were recorded as drug name, read v2, BNF and/or dm+d codes. Using the code lists provided by UK Biobank, together with recognised clinical/medication code repositories from other studies including CALIBER and clinicalcodes.org, we created anxiety and depression diagnosis and prescription code lists.

**Supplementary Table S2a: Anxiety diagnosis code list**

| **Read v2 code** | **CTV3 code** | **Description** |
| --- | --- | --- |
| 2258. |  | O/E - anxious |
| 225J. | XaKVA | O/E - panic attack |
| E200. |  | Anxiety states |
| E2000 |  | Anxiety state unspecified |
| E2001 | XE1Y7 | Panic disorder |
|  | Ua1qS |  |
| E2002 |  | Generalised anxiety disorder |
| E2003 | X00Sb | Anxiety with depression |
| E2004 |  | Chronic anxiety |
| E2005 |  | Recurrent anxiety |
| E200z |  | Anxiety state NOS |
| E205. | XE1YD | Neurasthenia - nervous debility |
|  | XE0qk |  |
|  |  | Nervous exhaustion |
| E28.. | XE1Ym | Acute reaction to stress |
|  | Xa18j |  |
| E280. |  | Acute panic state due to acute stress reaction |
| E283. |  | Other acute stress reactions |
| E283z |  | Other acute stress reaction NOS |
| E284. |  | Stress reaction causing mixed disturbance of emotion |
| E28z. | XE1Yn | Acute stress reaction NOS |
| Eu054 | X00RP | [X]Organic anxiety disorder |
| Eu341 | E2112 | [X]Dysthymia, [X]Persistant anxiety depression |
| Eu41. |  | [X]Other anxiety disorders |
| Eu410 |  | [X]Panic disorder [episodic paroxysmal anxiety] |
| Eu411 | E2002 E200. | [X]Generalized anxiety disorder |
| Eu412 | X00Sb | [X]Mixed anxiety and depressive disorder |
| Eu413 |  | [X]Other mixed anxiety disorders |
| Eu41y | XE1Zj | [X]Other specified anxiety disorders |
| Eu41z | E200. | [X]Anxiety disorder, unspecified |
| Eu430 | XE1Ym | [X]Acute stress reaction |

**Supplementary Table S2b: Depression diagnosis code list**

| **Read v2 code** | **CTV3 code** | **Read v2 Description** |
| --- | --- | --- |
| 1B17. | XE0re | Depressed |
|  |  |  |
|  |  |  |
|  | XM0CR |  |
|  | XM0CS |  |
|  | X00SO |  |
|  |  |  |
| 1BT.. | XE0re | Depressed mood |
| 2257. | 2257. | O/E - depressed |
| 62T1. | 62T1. | Puerperal depression |
| 9kQ.. | XaPKm | On full dose long term treatment depression - enh serv admin |
| E112. | XE1Y0 | Single major depressive episode |
|  | X00SQ |  |
|  | X00SR |  |
|  | X00SS |  |
| E1120 |  | Single major depressive episode, unspecified |
| E1121 |  | Single major depressive episode, mild |
| E1122 |  | Single major depressive episode, moderate |
| E1123 |  | Single major depressive episode, severe, without psychosis |
| E1124 |  | Single major depressive episode, severe, with psychosis |
| E1125 |  |  |
| E1126 |  |  |
| E112z |  | Single major depressive episode NOS |
| E113. | XE1Y1 | Recurrent major depressive episode |
|  | XM1GC |  |
| E1130 |  | Recurrent major depressive episodes, unspecified |
| E1131 |  | Recurrent major depressive episodes, mild |
| E1132 |  | Recurrent major depressive episodes, moderate |
| E1133 |  | Recurrent major depressive episodes, severe, no psychosis |
| E1134 |  | Recurrent major depressive episodes, severe, with psychosis |
| E1135 |  |  |
| E1136 |  |  |
| E1137 |  |  |
| E113z |  | Recurrent major depressive episode NOS |
| E11y2 |  | Atypical depressive disorder |
| E11z2 |  | Masked depression |
| E135. |  | Agitated depression |
| E2003 | X00Sb | Anxiety with depression |
| E204. | XE1YC | Neurotic depression reactive type |
| E291. |  | Prolonged depressive reaction |
| E2B.. |  | Depressive disorder NEC |
| E2B1. |  | Chronic depression |
| Eu32. | XE1Y0 | [X]Depressive episode |
|  | XE1Zb |  |
|  | XE1YC |  |
| Eu320 |  | [X]Mild depressive episode |
| Eu321 |  | [X]Moderate depressive episode |
| Eu322 | XE1ZY | [X]Severe depressive episode without psychotic symptoms |
|  | XaCHr |  |
|  | XaCHs |  |
| Eu323 | XE1ZZ | [X]Severe depressive episode with psychotic symptoms |
|  | E1124 |  |
| Eu324 | XaCIs | [X]Mild depression |
| Eu325 | XSGok | [X]Major depression, mild |
| Eu326 | XSGol | [X]Major depression, moderately severe |
| Eu327 | XSGom | [X]Major depression, severe without psychotic symptoms |
| Eu328 | XSGon | [X]Major depression, severe with psychotic symptoms |
| Eu329 | XaX53 | [X]Single major depr ep, severe with psych, psych in remiss |
| Eu32A | XaX54 | [X]Recurr major depr ep, severe with psych, psych in remiss |
| Eu32B | XaY2C | [X]Antenatal depression |
| Eu32y | XE1Za | [X]Other depressive episodes |
|  | X00SU |  |
| Eu32z | XE1Zb | [X]Depressive episode, unspecified |
|  | XE1YC |  |
|  | XaB9J |  |
| Eu33. | XE1Zc | [X]Recurrent depressive disorder |
|  | E1137 |  |
|  | E130. |  |
|  | X761L |  |
| Eu330 |  | [X]Recurrent depressive disorder, current episode mild |
| Eu331 |  | [X]Recurrent depressive disorder, current episode moderate |
| Eu332 | XE1Zd | [X]Recurr depress disorder cur epi severe without psyc sympt |
|  | E1133 |  |
|  | XaCHo |  |
| Eu333 | XE1Ze | [X]Recurrent depress disorder cur epi severe with psyc symp |
|  | E1134 |  |
| Eu334 |  | [X]Recurrent depressive disorder, currently in remission |
| Eu33y |  | [X]Other recurrent depressive disorders |
| Eu33z | XE1Zf | [X]Recurrent depressive disorder, unspecified |
|  | X00SO |  |
| Eu412 | X00Sb | [X]Mixed anxiety and depressive disorder |
| Eu530 | 62T1. | [X]Postnatal depression NOS |
| Eu34. | Eu34. | [X]Persistent mood affective disorders |
| Eu34y |  | [X]Other persistent mood affective disorders |
| Eu34z |  | [X]Persistent mood affective disorder, unspecified |
| Eu33z | XE1Zf | X]Recurrent depressive disorder, unspecified |
| Eu340 | E2113 | [X]Cyclothymia |
| Eu341 | E2112 | [X]Dysthymia |
| Eu3y. |  | [X]Other mood affective disorders |
| Eu3y0 | XE1Zg | [X]Other single mood affective disorders |
|  | Eu316 |  |
| Eu3y1 | XE1Zh | [X]Other recurrent mood affective disorders |
|  | Xa0wV |  |
| Eu3yy |  | [X]Other specified mood affective disorders |
| Eu3z. |  | [X]Unspecified mood affective disorder |
| E290. |  | Brief depressive reaction |
| E290z |  |  |

^1^ NHS Digital: Read codes [https://digital.nhs.uk/services/terminology-and-classifications/read-codes](https://digital.nhs.uk/services/terminology-and-classifications/read-codes)

**Supplementary Table S2c: Anti-anxiety/depressant prescription code list**

| Code |
| --- |
| CITALOPRAM da9.. CITALOPRAM da91. CITALOPRAM 20mg tablets da92. CIPRAMIL 20mg tablets da93. CITALOPRAM 10mg tablets da94. *CIPRAMIL 10mg tablets da95. CITALOPRAM 40mg tablets da96. *CIPRAMIL 40mg tablets da97. CIPRAMIL 40mg/mL oral drops 15mL da98. *PAXORAN 10mg tablets da99. *PAXORAN 20mg tablets da9A. *PAXORAN 40mg tablets da9z. CITALOPRAM 40mg/mL oral drops SERTRALINE da5.. SERTRALINE HYDROCHLORIDE da51. SERTRALINE 50mg tablets da52. SERTRALINE 100mg tablets da53. LUSTRAL 50mg tablets da54. LUSTRAL 100mg tablets FLUVOXAMINE da3.. FLUVOXAMINE MALEATE da31. FAVERIN 50mg tablets da32. FLUVOXAMINE MALEATE 50mg tablets da33. FAVERIN 100mg tablets da34. FLUVOXAMINE MALEATE 100mg tablets FLUOXETINE da4.. FLUOXETINE HYDROCHLORIDE da41. FLUOXETINE 20mg capsules da42. *PROZAC 20mg capsules x30 da43. FLUOXETINE 20mg/5mL oral liquid da44. PROZAC 20mg/5mL oral liquid da45. PROZAC 20mg capsules da46. FLUOXETINE 60mg capsules da47. *PROZAC 60mg capsules da48. *FELICIUM 20mg capsules da49. OXACTIN 20mg capsules da4A. RANFLUTIN 20mg capsules da4B. PROZIT 20mg/5mL oral solution da4C. PROZEP 20mg/5mL oral solution da4D. OLENA 20mg dispersible tablets da4E. FLUOXETINE 20mg dispersible tablets PAROXETINE da6.. PAROXETINE HYDROCHLORIDE da61. PAROXETINE 20mg tablets da62. SEROXAT 20mg tablets x30 da63. PAROXETINE 30mg tablets da64. SEROXAT 30mg tablets x30 da65. PAROXETINE 10mg/5mL sugar free liquid da66. SEROXAT 10mg/5mL sugar free liquid da67. PAROXETINE 10mg tablets da68. SEROXAT 10mg tablets MIRTAZAPINE daB.. MIRTAZAPINE daB1. MIRTAZAPINE 30mg tablets daB2. *ZISPIN 30mg tablets daB3. MIRTAZAPINE 30mg oro-dispersible tablets daB4. ZISPIN SOLTAB 30mg oro-dispersible tablets daB5. MIRTAZAPINE 15mg oro-dispersible tablets daB6. ZISPIN SOLTAB 15mg oro-dispersible tablets daB7. MIRTAZAPINE 45mg oro-dispersible tablets daB8. ZISPIN SOLTAB 45mg oro-dispersible tablets daBy. MIRTAZAPINE 45mg tablets daBz. MIRTAZAPINE 15mg tablets DOSULEPIN/DOTHIEPIN/ PROTHIADEN  d75.. DOSULEPIN HYDROCHLORIDE d751. PROTHIADEN 25mg capsules d752. PROTHIADEN 75mg tablets d753. *PREPADINE 25mg capsules d754. *PREPADINE 75mg tablets d755. *DOTHAPAX 25mg capsules d756. *DOTHAPAX 75mg tablets d759. *THADEN 25mg capsules d75A. *THADEN 75mg tablets d75y. DOSULEPIN HYDROCHLORIDE 25mg capsules d75z. DOSULEPIN HYDROCHLORIDE 75mg tablets LOFEPRAMINE d79.. LOFEPRAMINE d791. *GAMANIL 70mg tablets x56CP d792. LOMONT 70mg/5mL sugar free suspension d793. *FEPRAPAX 70mg tablets d794. *GAMANIL 70mg tablets d79y. LOFEPRAMINE 70mg/5mL sugar free suspension d79z. LOFEPRAMINE 70mg tablets VENLAFAXINE da7.. VENLAFAXINE da71. VENLAFAXINE 37.5mg tablets da72. VENLAFAXINE 75mg tablets da73. *EFEXOR 37.5mg tablets da74. *EFEXOR 75mg tablets da75. *VENLAFAXINE 50mg tablets da76. *EFEXOR 50mg tablets da77. VENLAFAXINE 75mg m/r capsules da78. EFEXOR XL 75mg m/r capsules da79. VENLAFAXINE 150mg m/r capsules da7a. VENAXX XL 150mg m/r capsules da7A. EFEXOR XL 150mg m/r capsules da7b. VAXALIN XL 75mg m/r capsules da7B. RODOMEL XL 75mg m/r capsules da7c. VAXALIN XL 150mg m/r capsules da7C. RODOMEL XL 150mg m/r capsules da7d. ALVENTA XL 75mg m/r capsules da7D. WINFEX XL 75mg m/r capsules da7e. ALVENTA XL 150mg m/r capsules da7E. WINFEX XL 150mg m/r capsules da7f. RANFAXINE XL 150mg m/r capsules da7F. TRIXAT XL 75mg m/r capsules da7g. RANFAXINE XL 75mg m/r capsules da7G. TRIXAT XL 150mg m/r capsules da7h. BONILUX XL 75mg m/r capsules da7H. VIEPAX XL 75mg m/r tablets da7i. BONILUX XL 150mg m/r capsules da7I. VENLAFAXINE 75mg m/r tablets da7j. TONPULAR XL 75mg m/r capsules da7J. VIEPAX XL 150mg m/r tablets da7k. TONPULAR XL 150mg m/r capsules da7K. VENLAFAXINE 150mg m/r tablets da7l. FORAVEN XL 75mg m/r capsules da7L. *TARDCAPS XL 75mg m/r capsules da7m. FORAVEN XL 150mg m/r capsules da7M. *TARDCAPS XL 150mg m/r capsule da7n. DEPEFEX XL 75mg m/r capsules da7N. VIEPAX 37.5mg tablets da7o. DEPEFEX XL 150mg m/r capsules da7O. VIEPAX 75mg tablets da7p. VENLALIC XL 37.5mg m/r tablets da7P. VENSIR XL 75mg m/r capsules da7q. VENLAFAXINE 37.5mg m/r tablets da7Q. VENSIR XL 150mg m/r capsules da7r. SUNVENIZ XL 75mg m/r tablets da7R. TIFAXIN XL 75mg m/r capsules da7s. SUNVENIZ XL 150mg m/r tablets da7S. TIFAXIN XL 150mg m/r capsules da7t. VENLADEX XL 75mg m/r tablets da7T. VEXARIN XL 75mg m/r capsules da7u. VENLADEX XL 150mg m/r tablets da7U. VEXARIN XL 150mg m/r capsules da7v. EFEXOR XL 225mg m/r capsules da7V. VENLALIC XL 75mg m/r tablets da7w. VENLAFAXINE 225mg m/r capsules da7W. VENLALIC XL 150mg m/r tablets da7X. VENLALIC XL 225mg m/r tablets da7Y. VENLAFAXINE 225mg m/r tablets da7Z. VENAXX XL 75mg m/r capsules ESCITALOPRAM daC.. ESCITALOPRAM daC1. ESCITALOPRAM 10mg tablets daC2. CIPRALEX 10mg tablets daC3. ESCITALOPRAM 20mg tablets daC4. CIPRALEX 20mg tablets daC5. ESCITALOPRAM 5mg tablets daC6. CIPRALEX 5mg tablets daC7. ESCITALOPRAM 10mg/mL oral drops daC8. *CIPRALEX 10mg/mL oral drops daC9. CIPRALEX 20mg/mL oral drops daCA. ESCITALOPRAM 20mg/mL oral drops |

**Supplementary Table S3. Incident cases post Biobank baseline and prevalent cases pre-biobank baseline adjusted for pre-existing anxiety/depression diagnosis or prescription, complete case analysis.**

**Cox proportional hazard models of the association between socio-demographic, health, lifestyle and work characteristics and incident anxiety/depression in a) all IT workers compared to all other employed Biobank participants b) IT worker subgroups (managers, professionals, technicians) and c) IT subgroups compared to other similar occupations within their SOC tree (Functional Managers, Science and Technology Professionals, Science and Technology Associate Professionals).** 

*Longitudinal study population: all employed Biobank participants with linked GP records* 

|  |  | **Model 0** ^d^ | **Model 1** ^e^ | **Model 2** ^f^ | **Model 3** ^g^ | **Model 4** ^h^ |
| --- | --- | --- | --- | --- | --- | --- |
|  |  | Unadjusted HR (95% CI) | Adjusted HR (95% CI) | Adjusted HR (95% CI) | Adjusted HR (95% CI) | Adjusted HR  (95% CI) |
| **a** | Failures 21,035 |  |  |  |  |  |
|  | All other employed participants Incidence rate* (2.4) All IT workers Incidence rate* (1.8) | 1.00 0.76 (0.70-0.84) | 1.00 0.85 (0.78-0.93) | 1.00 0.92 (0.84-1.01) | 1.00 0.93 (0.85-1.02) | 1.00 0.96 (0.88-1.05) |
| **b** | Failures 504 |  |  |  |  |  |
|  | IT managers Incidence rate* (1.8) IT professionals Incidence rate* (1.7) IT technicians Incidence rate* (2.2) | 1.00 0.98 (0.81-1.19) 1.24 (0.95-1.61) | 1.00 1.01 (0.84-1.23) 1.18 (0.91-1.54) | 1.00 0.95 (0.78-1.16) 0.99 (0.75-1.31) | 1.00 0.96 (0.79-1.18) 1.00 (0.76-1.32) | 1.00 0.97 (0.79-1.18) 0.95 (0.72-1.27) |
| **c** | Failures 767 |  |  |  |  |  |
|  | All other Functional managers Incidence rate* (1.9) IT managers Incidence rate* (1.8) | 1.00 0.94 (0.79-1.11) | 1.00 1.00 (0.84-1.18) | 1.00 1.00 (0.84-1.18) | 1.00 0.98 (0.83-1.16) | 1.00 1.03 (0.87-1.23) |
|  | Failures 576 |  |  |  |  |  |
|  | All other Science & Technology professionals Incidence rate* (1.4) IT professionals Incidence rate* (1.7) | 1.00 1.18 (1.00-1.39) | 1.00 1.08 (0.91-1.28) | 1.00 1.09 (0.92-1.29) | 1.00 1.07 (0.91-1.27) | 1.00 1.11 (0.92-1.35) |
|  | Failures 250 |  |  |  |  |  |
|  | All other Science & Technology Associate professionals Incidence rate* (1.9) IT technicians Incidence rate* (2.2) | 1.00 1.15 (0.88-1.50) | 1.00 1.04 0.80-1.36) | 1.00 1.05 (0.80-1.38) | 1.00 1.07 (0.81-1.40) | 1.00 1.09 (0.82-1.46) |

HR, hazard ratio; CI, confidence interval.

Model 0 ^d ^= Unadjusted

Model 1 ^e^ = Model 0 + sociodemographic factors i.e., confounders (age, sex, ethnicity, date of Biobank recruitment/baseline assessment, pre-existing anxiety/depression diagnosis or script)

Model 2 ^f^ = Model 1 + additional sociodemographic factors i.e., potential mediators (deprivation, annual household income, pre-existing anxiety/depression diagnosis or script)

Model 3 ^g^ = Model 2 + health and lifestyle factors (smoking, sleep duration, BMI, longstanding illness/disability, pre-existing anxiety/depression diagnosis or prescription).

Model 4 ^h^ = Model 3 + work-related factors (tenure in current occupation, working hours, work-related sedentary behaviour, shift work, pre-existing anxiety/depression diagnosis or prescription).

*Rates are expressed per 100 and based on person-years

**Supplementary Table S4. Incident cases only post biobank baseline, complete case analysis.**

**Cox proportional hazard models of the association between socio-demographic, health, lifestyle and work characteristics and incident anxiety/depression in a) all IT workers compared to all other employed Biobank participants b) IT worker subgroups (managers, professionals, technicians) and c) IT subgroups compared to other similar occupations within their SOC tree (Functional Managers, Science and Technology Professionals, Science and Technology Associate Professionals).** 

*Longitudinal study population: all employed Biobank participants with linked GP records* 

|  |  | **Model 0** ^d^ | **Model 1** ^e^ | **Model 2** ^f^ | **Model 3** ^g^ | **Model 4** ^h^ |
| --- | --- | --- | --- | --- | --- | --- |
|  |  | Unadjusted HR (95% CI) | Adjusted HR (95% CI) | Adjusted HR (95% CI) | Adjusted HR (95% CI) | Adjusted HR  (95% CI) |
| **a** | Failures 11,983 |  |  |  |  |  |
|  | All other employed participants Incidence rate* (2.2) All IT workers Incidence rate* (1.7) | 1.00 0.75 (0.68-0.83) | 1.00 0.84 (0.76-0.93) | 1.00 0.92 (0.83-1.02) | 1.00 0.93 (0.83-1.03) | 1.00 0.98 (0.88-1.09) |
| **b** | Failures 376 |  |  |  |  |  |
|  | IT managers Incidence rate* (1.7) IT professionals Incidence rate* (1.5) IT technicians Incidence rate* (2.0) | 1.00 0.86 (0.69-1.08) 1.17 (0.86-1.59) | 1.00 0.90 (0.72-1.12) 1.12 (0.82-1.53) | 1.00 0.84 (0.67-1.06) 0.95 (0.68-1.32) | 1.00 0.85 (0.67-1.06) 0.95 (0.68-1.32) | 1.00 0.86 (0.68-1.08) 0.89 (0.63-1.24) |
| **c** | Failures 578 |  |  |  |  |  |
|  | All other Functional managers Incidence rate* (1.8) IT managers Incidence rate* (1.7) | 1.00 0.98 (0.81-1.19) | 1.00 1.05 (0.86-1.27) | 1.00 1.05 (0.86-1.28) | 1.00 1.03 (0.85-1.26) | 1.00 1.08 (0.89-1.31) |
|  | Failures 404 |  |  |  |  |  |
|  | All other Science & Technology professionals Incidence rate* (1.3) IT professionals Incidence rate* (1.5) | 1.00 1.12 (0.92-1.36**)** | 1.00 1.03 (0.84-1.26) | 1.00 1.02 (0.84-1.25) | 1.00 1.01 (0.83-1.23) | 1.00 1.07 (0.86-1.35) |
|  | Failures 168 |  |  |  |  |  |
|  | All other Science & Technology Associate professionals Incidence rate* (1.7) IT technicians Incidence rate* (2.0) | 1.00 1.19 (0.87-1.64) | 1.00 1.10 (0.80-1.52) | 1.00 1.07 (0.78-1.49) | 1.00 1.09 (0.79-1.51) | 1.00 1.17 (0.82-1.67) |

HR, hazard ratio; CI, confidence interval.

Model 0 ^d ^= Unadjusted

Model 1 ^e^ = Model 0 + sociodemographic factors i.e., confounders (age, sex, ethnicity, date of Biobank recruitment/baseline assessment)

Model 2 ^f^ = Model 1 + additional sociodemographic factors i.e., potential mediators (deprivation, annual household income)

Model 3 ^g^ = Model 2 + health and lifestyle factors (smoking, sleep duration, BMI, longstanding illness/disability).

Model 4 ^h^ = Model 3 + work-related factors (tenure in current occupation, working hours, work-related sedentary behaviour, shift work).

*Rates are expressed per 100 and based on person-years

**Supplementary Table S5. Incident cases stratified by age "40-55" and "55+" year groups, complete case analysis.** 

**Cox proportional hazard models of the association between socio-demographic, health, lifestyle and work characteristics and incident anxiety/depression in a) all IT workers compared to all other employed Biobank participants b) IT worker subgroups (managers, professionals, technicians) and c) IT subgroups compared to other similar occupations within their SOC tree (Functional Managers, Science and Technology Professionals, Science and Technology Associate Professionals).** 

*Longitudinal study population: all employed Biobank participants with linked GP records* 

|  |  | **Model 0** ^d^ |  | **Model 1** ^e^ |  | **Model 2** ^f^ |  | **Model 3** ^g^ |  | **Model 4** ^h^ |  |
| --- | --- | --- | --- | --- | --- | --- | --- | --- | --- | --- | --- |
|  |  | Unadjusted HR (95% CI) |  | Adjusted HR (95% CI) |  | Adjusted HR (95% CI) |  | Adjusted HR (95% CI) |  | Adjusted HR  (95% CI) |  |
| **a** | **Age (years)** | **40-55** | **55+** | **40-55** | **55+** | **40-55** | **55+** | **40-55** | **55+** | **40-55** | **55+** |
|  | Failures 40-55 years Failures 55+ years | 8,414 3569 |  |  |  |  |  |  |  |  |  |
|  | All other employed participants Incidence rate 40-55 years* (2.5) Incidence rate 55+ years* (1.7) All IT workers Incidence rate 40-55 years* (1.8) Incidence rate 55+ years* (1.2) | 1.00 0.72 (0.64-0.81) | 1.00 0.67 (0.52-0.86) | 1.00 0.86 (0.77-0.97) | 1.00 0.76 (0.59- 0.98) | 1.00 0.95 (0.85-1.07) | 1.00 0.83 (0.64-1.06) | 1.00 0.96 (0.85-1.07) | 1.00 0.83 (0.64-1.06) | 1.00 1.01 (0.90- 1.14) | 1.00 0.86 (0.67-1.11) |
| **b** | Failures 40-55 years Failures 55+ years | 313 63 |  |  |  |  |  |  |  |  |  |
|  | IT managers Incidence rate 40-55 years* (1.9) Incidence rate 55+ years* (1.2) IT professionals Incidence rate 40-55 years* (1.7) Incidence rate 55+ years* (1.2) IT technicians Incidence rate 40-55 years* (2.4) Incidence rate 55+ years* (1.1) | 1.00 0.86 (0.62- 1.09) 1.28 (0.91-1.78) | 1.00 0.93 (0.54- 1.60) 0.91 (0.42-2.00) | 1.00 0.88 (0.69- 1.13) 1.18 (0.84-1.66) | 1.00 0.95 (0.55-1.65) 0.86 (0.39-1.88) | 1.00 0.84 (0.65- 1.08) 1.00 (0.70- 1.44) | 1.00 0.87 (0.49-1.54) 0.72 (0.32-1.63) | 1.00 0.85 (0.66-1.09) 1.00 (0.70-1.44) | 1.00 0.88 (0.50-1.56) 0.71 (0.31-1.62) | 1.00 0.87 (0.68-1.13) 0.95 (0.68 -1.38) | 1.00 0.87 (0.48-1.57) 0.54 (0.23-1.30) |
| **c** | Failures 40-55 years Failures 55+ years | 464 114 |  |  |  |  |  |  |  |  |  |
|  | All other Functional managers Incidence rate 40-55 years* (2.0) Incidence rate 55+ years* (1.3) IT managers Incidence rate 40-55 years* (1.9) Incidence rate 55+ years* (1.2) | 1.00 0.95 (0.77-1.18) | 1.00 0.95 (0.59-1.52) | 1.00 1.07 (0.86-1.33) | 1.00 0.95 (0.59- 1.53) | 1.00 1.07 (0.86-1.33) | 1.00 0.97 (0.60- 1.56) | 1.00 1.05 (0.85-1.31) | 1.00 0.94 (0.58-1.52) | 1.00 1.09 (0.88-1.36) | 1.00 0.93 (0.57-1.51) |
|  | Failures 40-55 years Failures 55+ years | 311 93 |  |  |  |  |  |  |  |  |  |
|  | All other Science & Technology professionals Incidence rate 40-55 years* (1.5) Incidence rate 55+ years* (0.99) IT professionals Incidence rate 40-55 years* (1.7) Incidence rate 55+ years* (1.2) | 1.00 1.04 (0.83-1.29) | 1.00 1.14 (0.75-1.74) | 1.00 1.02 (0.82 -1.28) | 1.00 1.06 (0.69-1.64) | 1.00 1.03 (0.82 -1.29) | 1.00 1.01 (0.65-1.57) | 1.00 1.01 (0.81-1.27) | 1.00 0.94 (0.60-1.48) | 1.00 1.08 (0.83-1.4) | 1.00 1.00 (0.61-1.64) |
|  | Failures 40-55 years Failures 55+ years | 121 47 |  |  |  |  |  |  |  |  |  |
|  | All other Science & Technology Associate professionals Incidence rate 40-55 years* (1.9) Incidence rate 55+ years* (1.4) IT technicians Incidence rate 40-55 years* (2.4) Incidence rate 55+ years* (1.1) | 1.00 1.30  (0.90-1.87) | 1.00 0.74 (0.36-1.54) | 1.00 1.25 (0.86-1.80) | 1.00 0.71 (0.34-1.48) | 1.00 1.26 (0.87-1.82) | 1.00 0.68 (0.33-1.44) | 1.00 1.26 (0.87 -1.83) | 1.00 0.84 (0.39-1.79) | 1.00 1.31 (0.87-1.95) | 1.00 1.04 (0.47-2.30) |

HR, hazard ratio; CI, confidence interval.

Model 0 ^d ^= Unadjusted

Model 1 ^e^ = Model 0 + sociodemographic factors i.e., confounders (age, sex, ethnicity, date of Biobank recruitment/baseline assessment)

Model 2 ^f^ = Model 1 + additional sociodemographic factors i.e., potential mediators (deprivation, annual household income)

Model 3 ^g^ = Model 2 + health and lifestyle factors (smoking, sleep duration, BMI, longstanding illness/disability).

Model 4 ^h^ = Model 3 + work-related factors (tenure in current occupation, working hours, work-related sedentary behaviour, shift work).

*Rates are expressed per 100 and based on person-years

**Supplementary Table S6. Incident cases** **for anxiety/depression diagnosis only, complete case analysis.**

**Cox proportional hazard models of the association between socio-demographic, health, lifestyle and work characteristics and incident anxiety/depression in a) all IT workers compared to all other employed Biobank participants b) IT worker subgroups (managers, professionals, technicians) and c) IT subgroups compared to other similar occupations within their SOC tree (Functional Managers, Science and Technology Professionals, Science and Technology Associate Professionals).** 

*Longitudinal study population: all employed Biobank participants with linked GP records* 

|  |  | **Model 0** ^d^ | **Model 1** ^e^ | **Model 2** ^f^ | **Model 3** ^g^ | **Model 4** ^h^ |
| --- | --- | --- | --- | --- | --- | --- |
|  | **ANXIETY/DEPRESSION DIAGNOSIS ONLY** |  |  |  |  |  |
|  |  | Unadjusted HR (95% CI) | Adjusted HR (95% CI) | Adjusted HR (95% CI) | Adjusted HR (95% CI) | Adjusted HR  (95% CI) |
| **a** | Failures 12,136 |  |  |  |  |  |
|  | All other employed participants Incidence rate* (1.6) All IT workers Incidence rate* (1.3) | 1.00 0.79 (0.70-0.89) | 1.00 0.87 (0.77-0.97) | 1.00 0.96 (0.85-1.08) | 1.00 0.96 (0.86-1.09) | 1.00 1.01 (0.90-1.14) |
| **b** | Failures 294 |  |  |  |  |  |
|  | IT managers Incidence rate* (1.3) IT professionals Incidence rate* (1.2) IT technicians Incidence rate* (1.4) | 1.00 0.92 (0.71-1.18) 1.14 (0.80-1.63) | 1.00 0.96 (0.74-1.23) 1.09 (0.76-1.56) | 1.00 0.90 (0,69-1.16) 0.93 (0.64-1.36) | 1.00 0.90 (0.69-1.17) 0.93 (0.64-1.36) | 1.00 0.91 (0.70-1.19) 0.86 (0.58-1.26) |
| **c** | Failures 431 |  |  |  |  |  |
|  | All other Functional managers Incidence rate* (1.3) IT managers Incidence rate* (1.3) | 1.00 1.01 (0.81-1.26) | 1.00 1.07 (0.85-1.34) | 1.00 1.06 (0.85-1.33) | 1.00 1.05 (0.83-1.31) | 1.00 1.08 (0.86-1.36) |
|  | Failures 318 |  |  |  |  |  |
|  | All other Science & Technology professionals Incidence rate* (0.9) IT professionals Incidence rate* (1.2) | 1.00 1.18 (0.94-1.47) | 1.00 1.08 (0.86-1.35) | 1.00 1.07 (0.86-1.34) | 1.00 1.06 (0.85-1.33) | 1.00 1.14 (0.88-1.47) |
|  | Failures 112 |  |  |  |  |  |
|  | All other Science & Technology Associate professionals Incidence rate* (1.1) IT technicians Incidence rate* (1.4) | 1.00 1.41 (0.97-2.07) | 1.00 1.28 (0.87-1.88) | 1.00 1.26 (0.85-1.86) | 1.00 1.29 (0.87-1.92) | 1.00 1.26 (0.82-1.93) |

HR, hazard ratio; CI, confidence interval.

Model 0 ^d ^= Unadjusted

Model 1 ^e^ = Model 0 + sociodemographic factors i.e., confounders (age, sex, ethnicity, date of Biobank recruitment/baseline assessment)

Model 2 ^f^ = Model 1 + additional sociodemographic factors i.e., potential mediators (deprivation, annual household income)

Model 3 ^g^ = Model 2 + health and lifestyle factors (smoking, sleep duration, BMI, longstanding illness/disability).

Model 4 ^h^ = Model 3 + work-related factors (tenure in current occupation, working hours, work-related sedentary behaviour, shift work).

*Rates are expressed per 100 and based on person-years

**Supplementary Table S7. Incident cases for anxiety/depression prescriptions only, complete case analysis.**

**Cox proportional hazard models of the association between socio-demographic, health, lifestyle and work characteristics and incident anxiety/depression in a) all IT workers compared to all other employed Biobank participants b) IT worker subgroups (managers, professionals, technicians) and c) IT subgroups compared to other similar occupations within their SOC tree (Functional Managers, Science and Technology Professionals, Science and Technology Associate Professionals).** 

*Longitudinal study population: all employed Biobank participants with linked GP records* 

|  |  | **Model 0** ^d^ | **Model 1** ^e^ | **Model 2** ^f^ | **Model 3** ^g^ | **Model 4** ^h^ |
| --- | --- | --- | --- | --- | --- | --- |
|  | **ANXIETY/DEPRESSION PRESCRIPTIONS ONLY** |  |  |  |  |  |
|  |  | Unadjusted HR (95% CI) | Adjusted HR (95% CI) | Adjusted HR (95% CI) | Adjusted HR (95% CI) | Adjusted HR  (95% CI) |
| **a** | Failures 9,680 |  |  |  |  |  |
|  | All other employed participants Incidence rate* (1.7) All IT workers Incidence rate* (1.3) | 1.00 0.70 (0.62-0.78) | 1.00 0.78 (0.70-0.88) | 1.00 0.87 (0.77-0.98) | 1.00 0.87 (0.77-0.98) | 1.00 0.92 (0.81-1.04) |
| **b** | Failures 282 |  |  |  |  |  |
|  | IT managers Incidence rate* (1.4) IT professionals Incidence rate* (1.1) IT technicians Incidence rate* (1.6) | 1.00 0.73 (0.57-0.94) 1.14 (0.80-1.60) | 1.00 0.76 (0.59-0.99) 1.09 (0.77-1.54) | 1.00 0.71 (0.55-0.92) 0.91 (0.62-1.31) | 1.00 0.71 (0.55-0.93) 0.91 (0.63-1.32) | 1.00 0.72 (0.55-0.94) 0.85 (0.58-1.24) |
| **c** | Failures 439 |  |  |  |  |  |
|  | All other Functional managers Incidence rate* (1.3) IT managers Incidence rate* (1.4) | 1.00 1.07 (0.86-1.33) | 1.00 1.16 (0.93-1.44) | 1.00 1.16 (0.93-1.44) | 1.00 1.14  (0.91-1.42) | 1.00 1.17 (0.93-1.46) |
|  | Failures 292 |  |  |  |  |  |
|  | All other Science & Technology professionals Incidence rate* (1.0) IT professionals Incidence rate* (1.1) | 1.00 1.03 (0.82-1.30) | 1.00 0.95 (0.75-1.20) | 1.00 0.95 (0.75-1.20) | 1.00 0.94 (0.74-1.18) | 1.00 1.02 (0.78-1.34) |
|  | Failures 136 |  |  |  |  |  |
|  | All other Science & Technology Associate professionals Incidence rate* (1.4) IT technicians Incidence rate* (1.6) | 1.00 1.15 (0.81-1.64) | 1.00 1.08 (0.75-1.54) | 1.00 1.06 (0.73-1.52) | 1.00 1.08 (0.75-1.56) | 1.00 1.21 (0.82-1.79) |

HR, hazard ratio; CI, confidence interval.

Model 0 ^d ^= Unadjusted

Model 1 ^e^ = Model 0 + sociodemographic factors i.e., confounders (age, sex, ethnicity, date of Biobank recruitment/baseline assessment)

Model 2 ^f^ = Model 1 + additional sociodemographic factors i.e., potential mediators (deprivation, annual household income)

Model 3 ^g^ = Model 2 + health and lifestyle factors (smoking, sleep duration, BMI, longstanding illness/disability).

Model 4 ^h^ = Model 3 + work-related factors (tenure in current occupation, working hours, work-related sedentary behaviour, shift work).

*Rates are expressed per 100 and based on person-years

**Supplementary Table S8. Incident cases stratified by tenure in employment at baseline < 10 years and ≥ 10 years, complete case analysis.**

**Cox proportional hazard models of the association between socio-demographic, health, lifestyle and work characteristics and incident anxiety/depression in a) all IT workers compared to all other employed Biobank participants b) IT worker subgroups (managers, professionals, technicians) and c) IT subgroups compared to other similar occupations within their SOC tree (Functional Managers, Science and Technology Professionals, Science and Technology Associate Professionals).** 

*Longitudinal study population: all employed Biobank participants with linked GP records* 

|  |  | **Model 0** ^d^ |  | **Model 1** ^e^ |  | **Model 2** ^f^ |  | **Model 3** ^g^ |  | **Model 4** ^h^ |  |
| --- | --- | --- | --- | --- | --- | --- | --- | --- | --- | --- | --- |
|  |  | Unadjusted HR (95% CI) |  | Adjusted HR (95% CI) |  | Adjusted HR (95% CI) |  | Adjusted HR (95% CI) |  | Adjusted HR  (95% CI) |  |
| **a** | **Tenure in employment (years)** | **< 10** | **≥ 10** | **< 10** | **≥ 10** | **< 10** | **≥ 10** | **< 10** | **≥ 10** | **< 10** | **≥ 10** |
|  | Failures < 10 years Failures ≥ 10 years | 7,113 4,870 |  |  |  |  |  |  |  |  |  |
|  | All other employed participants Incidence rate < 10 years* (2.4) Incidence rate ≥ 10 years* (1.9) All IT workers Incidence rate < 10 years* (1.8) Incidence rate ≥ 10 years* (1.6) | 1.00 0.74 (0.65- 0.85) | 1.00 0.78 (0.66- 0.90) | 1.00 0.85 (0.74-0.98) | 1.00 0.84 (0.72-0.98) | 1.00 0.95 (0.83-1.10) | 1.00 0.89 (0.77-1.04) | 1.00 0.95 (0.83-1.10) | 1.00 0.90 (0.77-1.06) | 1.00 0.99 (0.86-1.14) | 1.00 0.97 (0.83-1.13) |
| **b** | Failures < 10years Failures ≥ 10 years | 207 169 |  |  |  |  |  |  |  |  |  |
|  | IT managers Incidence rate < 10 years* (1.8) Incidence rate ≥ 10 years* (1.7) IT professionals Incidence rate < 10 years* (1.6) Incidence rate ≥ 10 years* (1.5) IT technicians Incidence rate < 10 years* (2.9) Incidence rate ≥ 10 years* (1.4) | 1.00 0.90 (0.66-1.22) 1.67 (1.12-2.50) | 1.00 0.83 (0.60-1.15) 0.79 (0.48-1.28) | 1.00 0.94 (0.69-1.27) 1.50 (1.00-2.26) | 1.00 0.84 (0.61-1.17) 0.79 (0.49-1.29) | 1.00 0.87 (0.64-1.19) 1.26 (0.81-1.95) | 1.00 0.79 (0.57-1.11) 0.68 (0.40-.1.13) | 1.00 0.87 (0.63- 1.19) 1.23 (0.79-1.90) | 1.00 0.80 (0.57-1.13) 0.69 (0.41-1.15) | 1.00 0.87 (0.63-1.20) 1.13 (0.72-1.77) | 1.00 0.82 (0.58-1.16) 0.65 (0.38-1.10) |
| **c** | Failures < 10years Failures ≥ 10 years | 346 232 |  |  |  |  |  |  |  |  |  |
|  | All other Functional managers Incidence rate < 10 years* (1.9) Incidence rate ≥ 10 years* (1.7) IT managers Incidence rate < 10 years* (1.8) Incidence rate ≥ 10 years* (1.7) | 1.00 0.95 (0.73-1.24) | 1.00 1.02 (0.77-1.36) | 1.00 1.06 (0.81-1.38) | 1.00 1.04 (0.78-1.39) | 1.00 1.07 (0.82-1.40) | 1.00 1.03 (0.77-1.38) | 1.00 1.04 (0.80-1.37) | 1.00 1.02 (0.76-1.37) | 1.00 1.07 (0.81-1.40) | 1.00 1.05 (0.78-1.42) |
|  | Failures < 10years Failures ≥ 10 years | 225 179 |  |  |  |  |  |  |  |  |  |
|  | All other Science & Technology professionals Incidence rate < 10 years* (1.5) Incidence rate ≥ 10 years* (1.1) IT professionals Incidence rate < 10 years* (1.6) Incidence rate ≥ 10 years* (1.5) | 1.00 1.03 (0.79-1.34) | 1.00 1.23 (0.91 -1.65) | 1.00 0.93 (0.71-1.22) | 1.00 1.15 (0.86-1.55) | 1.00 0.93 (0.71-1.22) | 1.00 1.12 (0.83-1.50) | 1.00 0.90 (0.69-1.18) | 1.00 1.12 (0.83-1.51) | 1.00 0.94 (0.70-1.27) | 1.00 1.23 (0.87-1.74) |
|  | Failures < 10years Failures ≥ 10 years | 97 71 |  |  |  |  |  |  |  |  |  |
|  | All other Science & Technology Associate professionals Incidence rate < 10 years* (1.8) Incidence rate ≥ 10 years* (1.6) IT technicians Incidence rate < 10 years* (2.9) Incidence rate ≥ 10 years* (1.4) | 1.00 1.68 (1.11-2.54) | 1.00 0.81 (0.49-1.34) | 1.00 1.51 (1.00-2.29) | 1.00 0.76 (0.46-1.27) | 1.00 1.37 (0.89-2.10) | 1.00 0.77 (0.46-1.29) | 1.00 1.41 (0.92-2.16) | 1.00 0.76 (0.45-1.28) | 1.00 1.49 (0.94-2.38) | 1.00 0.78 (0.45-1.37) |

HR, hazard ratio; CI, confidence interval.

Model 0 ^d ^= Unadjusted

Model 1 ^e^ = Model 0 + sociodemographic factors i.e., confounders (age, sex, ethnicity, date of Biobank recruitment/baseline assessment)

Model 2 ^f^ = Model 1 + additional sociodemographic factors i.e., potential mediators (deprivation, annual household income)

Model 3 ^g^ = Model 2 + health and lifestyle factors (smoking, sleep duration, BMI, longstanding illness/disability).

Model 4 ^h^ = Model 3 + work-related factors (tenure in current occupation, working hours, work-related sedentary behaviour, shift work).

*Rates are expressed per 100 and based on person-years
